# Complementary vertebrate *Wac* models exhibit phenotypes relevant to DeSanto-Shinawi Syndrome

**DOI:** 10.7554/eLife.109104

**Published:** 2026-09-03

**Authors:** Kang-Han Lee, April M Stafford, Maria Pacheco-Vergara, Karol Cichewicz, Cesar P Canales, Nicolas Seban, Melissa Corea, Darlene Rahbarian, Kelly E Bonekamp, Grant R Gillie, Dariangelly Pacheco-Cruz, Alyssa M Gill, Hye-Eun Hwang, Yeong-Eun Kim, Katie L Uhl, Tara E Jager, Marwan Shinawi, Xiaopeng Li, Andre Obenaus, Shane R Crandall, Juhee Jeong, Alex S Nord, Cheol-Hee Kim, Daniel Vogt

**Affiliations:** 1 Department of Biology, Chungnam National University Daejeon Republic of Korea; 2 Department of Pediatrics and Human Development, College of Human Medicine, Michigan State University Grand Rapids United States; 3 Department of Molecular Pathology, New York University College of Dentistry New York United States; 4 Department of Psychiatry and Behavioral Sciences, University of California Davis Davis United States; 5 Department of Neurobiology, Physiology and Behavior, University of California Davis Davis United States; 6 Department of Physiology, Michigan State University East Lansing United States; 7 Neuroscience Program, Michigan State University East Lansing United States; 8 Corewell Health Grand Rapids United States; 9 Division of Genetics and Genomic Medicine, Department of Pediatrics, Washington University School of Medicine St. Louis United States; 10 Director, Preclinical and Translational Imaging Center, School of Medicine, University of California Irvine Irvine United States; Duke University United States; Stanford University United States

**Keywords:** Wac, DESSH, GABA, Mouse, Zebrafish

## Abstract

Monogenic syndromes are associated with neurodevelopmental changes that result in cognitive impairments and neurobehavioral phenotypes, including autism and seizures. Limited studies and resources are available to make meaningful headway into the underlying molecular mechanisms that result in these symptoms. One such example is DeSanto-Shinawi Syndrome (DESSH), a rare disorder caused by pathogenic variants in the *WAC* gene. Individuals with DESSH syndrome exhibit a recognizable craniofacial gestalt, developmental delay/intellectual disability, neurobehavioral symptoms that include autism, ADHD, behavioral difficulties, and seizures. However, no thorough studies from a vertebrate model exist to understand how these changes occur. To overcome this, we developed both murine and zebrafish *Wac/wac* deletion mutants and studied whether their phenotypes recapitulate those described in individuals with DESSH syndrome. We first show that the two *Wac* models exhibit craniofacial and behavioral changes, reminiscent of abnormalities found in DESSH syndrome. In addition, each model revealed impacts on GABAergic neurons and further studies showed that the mouse mutants are susceptible to seizures, changes in brain volumes that are different between sexes and relevant behaviors. Finally, we uncovered transcriptional impacts of *Wac* loss-of-function in mice that will pave the way for future molecular studies into DESSH. These studies present two new vertebrate models that begin to uncover biological underpinnings of DESSH syndrome and elucidate the biology of *Wac*.

## Introduction

Pathogenic variants in the WW domain-containing adaptor with coiled coil, *Wac*, gene cause a neurodevelopmental disorder called DeSanto-Shinawi (DESSH) syndrome (aka WAC-related disorder) (DeSanto et al., 2015). DESSH syndrome is characterized by a constellation of developmental delay/intellectual disability, craniofacial dysmorphism, hypotonia, seizure, gastrointestinal problems (e.g. constipation and gastroesophageal reflux), and ophthalmological abnormalities (e.g. refractive errors and strabismus) (DeSanto et al., 2015). In addition, individuals with DESSH syndrome exhibit arrays of neurobehavioral phenotypes, including aggression, attention deficit hyperactivity disorder (ADHD), autism spectrum disorder (ASD) and anxiety (Alawadhi et al., 2021; DeSanto et al., 2015; Leonardi et al., 2020; Morales et al., 2022; Vanegas et al., 2018). *Wac* is a known autism risk gene due to the enrichment of pathogenic genetic variants in ASD cohorts (Iossifov et al., 2014). However, little is known about the function of WAC in the brain and whether suitable vertebrate models could explore the underlying biology of DESSH syndrome phenotypes.

The *Wac* gene encodes a protein with WW and coiled-coil domains, common protein/protein interaction regions. A recent study assessed the role of evolutionarily conserved protein domains in the human WAC protein that uncovered an amino-terminal nuclear localization signal as well as a highly conserved disorganized region that contains several putative phosphorylation motifs that are mutated in humans with known *WAC* variants (Rudolph et al., 2023). While the functional role of these domains and of human *WAC* itself are still poorly understood in vertebrates, a multitude of roles for the WAC protein have been proposed, including positive regulation of mammalian target of rapamycin (mTOR) signaling, mitosis, transcription, and autophagy (David-Morrison et al., 2016; McKnight et al., 2012; Qi et al., 2018; Zhang and Yu, 2011), which were worked out in *drosophila* and immortalized cell lines. However, whether these roles are conserved in vertebrates and their relevance to human DESSH symptoms is still unknown.

To better understand *Wac*’s role in vertebrates and in particular brain-relevant changes, we generated two species-specific vertebrate deletion models to assess whether *Wac* depletion could recapitulate symptoms associated with DESSH syndrome. The rationale for choosing mouse and zebrafish models were: (1) Both are vertebrates; (2) Unique genetic approaches in each model; (3) Species-specific phenotypic screening advantages; (4) Finally, utilizing two distinct vertebrate models to study key brain phenotypes will be a powerful means to uncover the fundamental biology of DESSH syndrome in a manner that is likely to reveal novel neurological underpinnings.

Overall, our models revealed some similarities in core DESSH symptoms, including craniofacial dysmorphism and relevant behaviors implicating decreased learning and social interaction. Notably, while each model displayed common decreased GABAergic markers, there were slight differences between species in targets. Additionally, mice had increased seizure susceptibility, while zebrafish did not; mice had only minor changes to sociability while zebrafish showed a strong change. We further took advantage of the mouse model, which more closely resembles humans, to probe into potential unexplored phenotypes and molecular changes. Despite some differences, both models revealed that males were more susceptible in multiple phenotypes and that male mice exhibited novel molecular changes that could be further investigated. The first vertebrate disease models for DESSH syndrome are presented here, and the multiple shared and unique phenotypes that could begin to inform the underlying biological mechanisms of DESSH.

## Results

### Validation of murine *Wac* and zebrafish *wac* mutants

**Figure 1.**
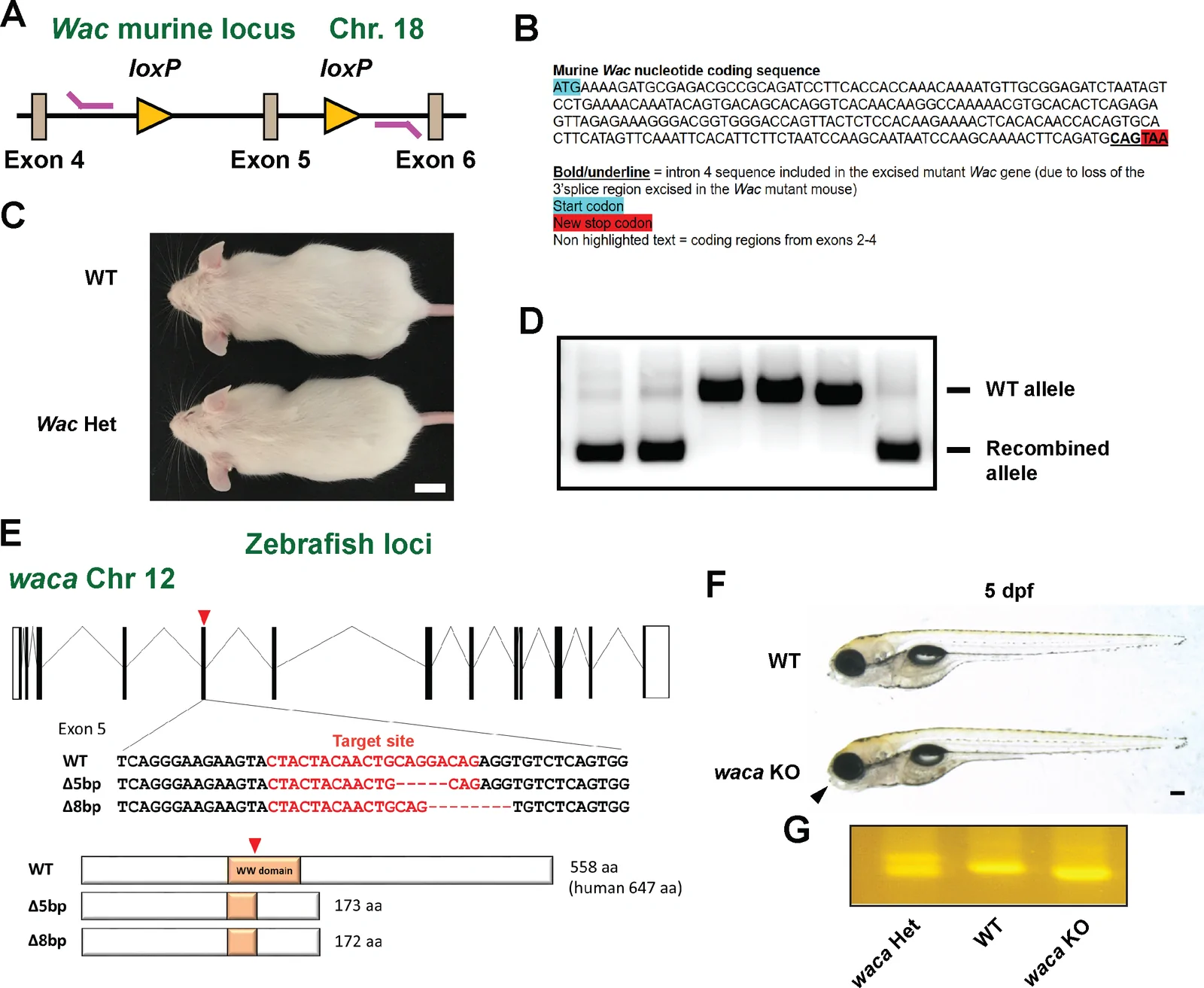
Generation and validation of murine *Wac* and zebrafish *waca* mutants. (**A**) Floxed mouse *Wac* genetic locus and genotyping primers. Genotyping primers (magenta lines) reside external to the loxP sites (orange triangles) flanking exon 5 and nearby introns. (**B**) The recombined DNA band was Sanger-sequenced and results show the start codon (blue) and the novel stop codon introduced by the frameshift mutation resulting from Cre-mediated deletion of the locus used to generate the locus. (**C**) Example images of adult wild-type (WT) and *Wac* Het mice. (**D**) DNA gel of genotyping for WT and recombined (Het) *Wac* alleles; upper band is ~1.5 kilobases and the lower band is ~500 basepairs. (**E**) *waca* KO zebrafish were generated by CRISPR/Cas9. KO target sites for sgRNAs are indicated by red arrowheads and predicted protein structures for KO mutations are indicated below the panel. (**F**) WT and *waca* KO zebrafish have grossly normal sizes but *waca* KO exhibited a shortened jaw (black triangle) at 5 dpf. (**G**) Example *waca* genotyping results showing a 116 bp for WT (upper band) and a 111 (lower band) bp for KO. Abbreviations: (aa) amino acid; (bp) base pair; (Chr) chromosome; (dpf) days post fertilization; (WT) wild-type. Scale bars (**C**) = 1 cm and (**F**) = 200 µm. Figure 1—source data 1.All genotyping. Figure 1—source data 2.Mouse Wac genotyping. Figure 1—source data 3.Zebrafish genotype.

We obtained sperm from the International Mouse Phenotyping Consortium (IMPC); the *Wac* murine locus on chromosome 18 was genetically modified to include flanking *loxP* (Flox) sites of exon 5 (Figure 1A). Sanger sequencing of the genotyped PCR validated the locus and an introduced frameshift that resulted in a premature stop codon (Figure 1B). *Wac flox* founders were bred and crossed to *beta-actin-Cre* mice, which express *Cre* in germ cells (Lewandoski et al., 1997). We generated wild-type (WT) and constitutive heterozygous (Het) progeny of similar size (Figure 1C) but not any live homozygous knockouts. Findings were consistent with data from the IMPC; constitutive *Wac* KOs are embryonic lethal. Results of genotyping using PCR are shown in Figure 1D. WAC protein was also reduced by 64% in Het brains, (shown later in Figure 5).

**Figure 1—figure supplement 1.**
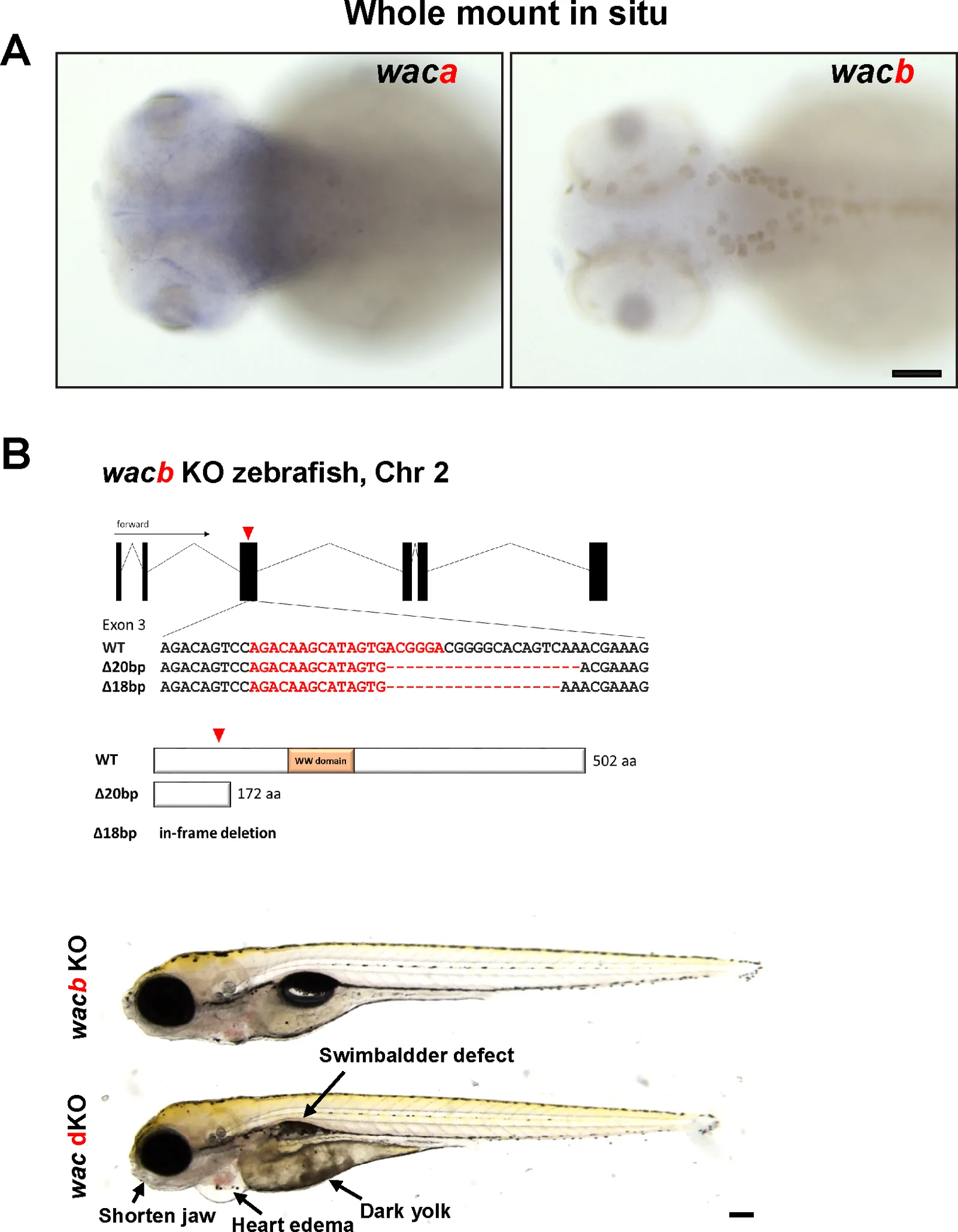
*waca* and *wacb* mRNA expression and generation of other zebrafish *wac* models. (**A**) Whole-mount in situ hybridization analysis of *waca* and *wacb* mRNA in zebrafish at 72 hr post fertilization. *wacb* KO zebrafish were generated by CRISPR/Cas9; double KOs were generated by intercrossing *waca* and *wacb* zebrafish. KO target site for sgRNA is indicated on the gene structure (arrowhead). Predicted protein structures show premature truncation caused by frameshift deletion mutations (–20 bp for *wacb* KO). (**B**) Morphological characterization of *wacb* KO and *waca,b* dKOs at 5 dpf. *wacb KO* zebrafish showed no obvious phenotypes at any stage analyzed, while *waca,wacb* dKO showed heart edema, dark yolk, and swimbladder defect in addition to shortened jaw. *waca,wacb* dKO zebrafish did not survive over 7 dpf. Scale bar in both (**A**) and (**B**) = 200 µm.

Zebrafish harbor two *wac* genes on chromosome 12, *waca*, and 2, *wacb*. A CRISPR/CAS9 approach was used to target these loci to create *waca* and *wacb* KOs and then interbreed to generate *waca; wacb* double KOs. Notably, *waca* expression was more widespread and higher in the brain of zebrafish compared to *wacb* (Figure 1—figure supplement 1), indicating that *waca* might be more important for DESSH. The *waca* mutant had a deletion in coding exon five (Figure 1E) with *waca* progeny grossly normal in size by 5 days post fertilization (dpf) but with a shortened lower jaw (Figure 1F); genotyping results are shown in Figure 1G. The *wacb* KO and *waca;wacb* double KO zebrafish are shown in Figure 1—figure supplement 1. Since the *wacb* KOs did not show phenotypes like the *waca* mutants, we did not report further data. Moreover, since double KOs exhibited early lethality, we chose to solely focus on *waca* KOs for this report. Some possible causes of lethality are denoted in Figure 1—figure supplement 1, with double KOs showing evidence of heart edema and loss of the swim bladder.

### Craniofacial changes in murine and zebrafish mutants

**Figure 2.**
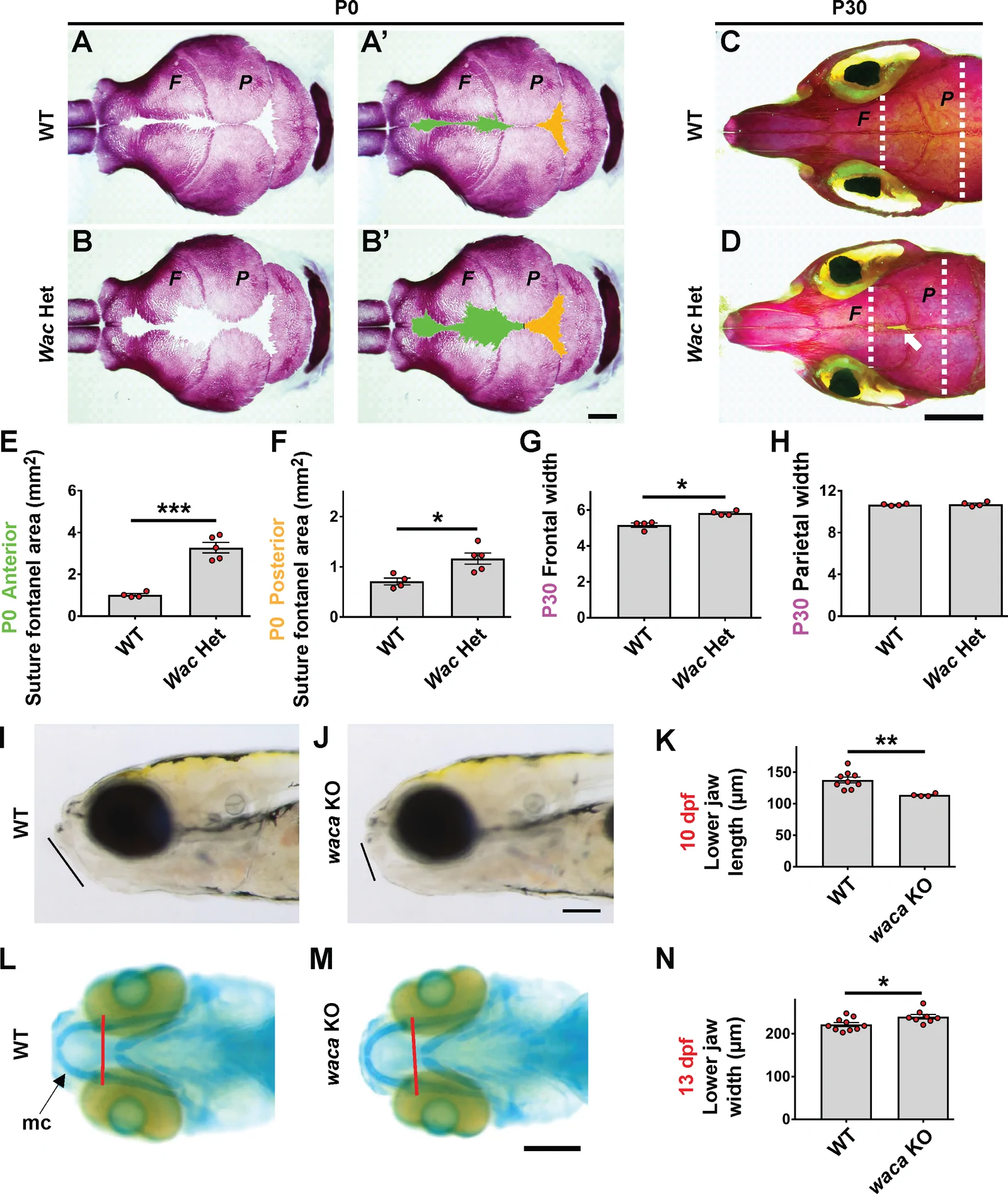
Craniofacial changes in mutants. (**A, B**) Dorsal views of the P0 calvaria stained with Alizarin red for bone. (**A’, B’**) The suture and fontanel areas in (**A**) and (**B**) are pseudo-colored green (anterior) and orange (posterior). (**C, D**) Dorsal views of P30 wild-type (WT) and *Wac* Het skull bones; arrow (**D**) points to a gap in the interfrontal suture. (**F**) Frontal bone and (**P**) Parietal bone; white dashed lines are widths measured in (**G, H**). (**E, F**) Quantification of P0 fontanel suture areas pseudo colored in A’ and B’; WT n=4, *Het* n=5. Quantification of the skull width across the frontal (**G**) and parietal (**H**) bones at P30 in WTs and *Wac* Hets; WT n=4, Het n=4. (**I, J**) The *waca* KO zebrafish show a shortened jaw structure, compared to WT. Lines denote lower jaw length. (**K**) Quantification of lower jaw length; n=9 WT and 4 *waca* KO. (**L, M**) Cartilage staining of zebrafish using alcian blue, ventral view. (**N**) Measurements of the width of Meckel’s cartilage in *waca* KO zebrafish at 13 dpf (red line); WT n=10 and KO n=8. Data are expressed as the mean ± SEM. *p<0.05, **p<0.01, and ***p<0.001. Scale bars (**B’**) = 1 mm, (**D**) = 4 mm, (**J, M**) = 200 µm.

Craniofacial dysmorphism is a cardinal feature in patients with DESSH syndrome (Alsahlawi et al., 2020; DeSanto et al., 2015; Hamdan et al., 2014; Ho et al., 2022; Leonardi et al., 2020; Lugtenberg et al., 2016; Morales et al., 2022; Uehara et al., 2018; Vanegas et al., 2018), with larger frontal facial features being common, including larger foreheads. We first wondered whether a reduction in *Wac* dosage in mice could recapitulate this finding. Postnatal day (P)0 and P30 mouse skulls were examined. During neonatal ages, a widening of the fontanels and sutures along the midline of the calvaria was noted (Figure 2A and B). While both the anterior and posterior regions of the calvaria were affected, the anterior region had the greater change (Figure 2A, B, E and F with pseudo-colored areas in 2 A’, B’; anterior *p*=0.0001, posterior *p*=0.01). By P30, there remained a noticeable gap in the interfrontal suture of the *Wac* Het. Concomitantly, the width of the skull across the frontal bones was significantly increased in *Wac* Hets, while the width across the parietal bones was normal (Figure 2C, D, G and H, *p*=0.002, frontal width). A feature that was notable was a shortened lower jaw in the *waca* KO zebrafish compared to WTs at 10 dpf (Figure 2I–K, *p*=0.006), suggesting similar abnormalities in frontal craniofacial structures. In addition, the *waca* mutants also exhibited changes in other frontal craniofacial features, including an increased width spanning Meckel’s cartilage at 13 dpf (Figure 2L–N, *p*=0.02). The *waca* KO zebrafish survived into adulthood and continued to show morphological craniofacial defects of shortened lower jaw, sunken head, and broad nasal tip at adult stage (data not shown). Thus, both models exhibit craniofacial changes relevant to DESSH syndrome.

### Behavioral changes in *Wac/wac* mutants

**Figure 3.**
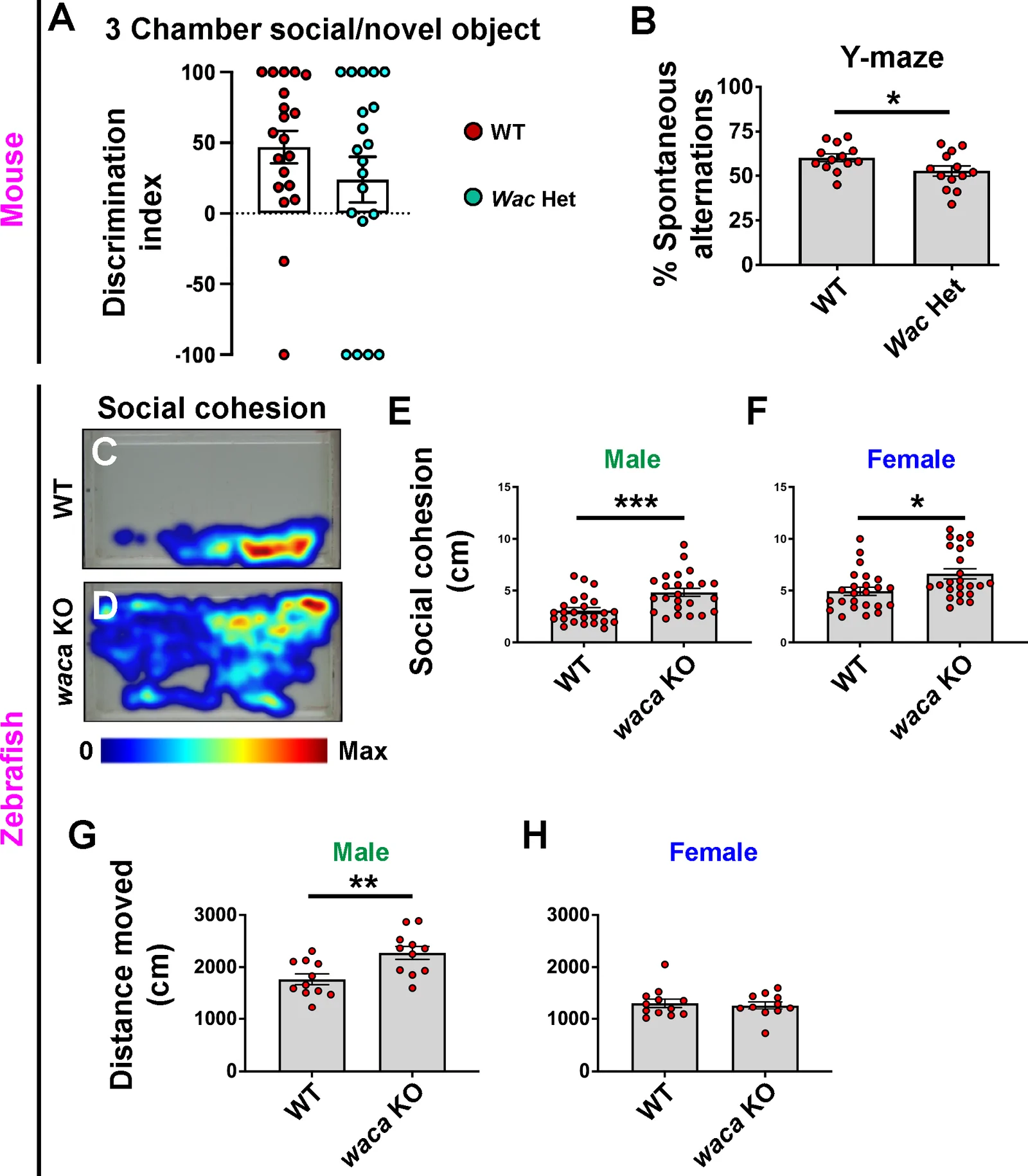
Behavioral changes in mutants. (**A**) Six to eight week WT and *Wac* Het mice were tested in the 3-chamber social/novel object test, wild-type (WT) n=20 and Het n=20, and the Y-maze (**B**), n=13 for both groups, with *Wac* Hets showing deficits in the Y-maze. (**C, D**) Example heat maps during the 17–19 min timeframe of the social cohesion test in zebrafish; *waca* KO males and females were more dispersed (**E, F**), see methods for test details. (**G, H**) Distance moved was greater in male *waca* KOs in the novel tank assay, n=11, both groups, compared to females, n=12 (WT) and 11 (KOs). Data are expressed as the mean ± SEM; *p<0.05, **p<0.01, and ***p<0.001.

Loss of *Wac* may impact social/cognitive behavior, as seen among individuals diagnosed with DESSH syndrome and as previous models exhibit intellectual challenges (Alsahlawi et al., 2020; Leonardi et al., 2020; Lugtenberg et al., 2016; Morales et al., 2022; Uehara et al., 2018). We noticed that Het mutant mice had normal ambulation, total distance traveled, and time spent in different areas of the open field (Figure 3—figure supplement 1). In a 3-chamber social test, while some *Wac* Hets trended towards less social discrimination using a previously described test (Phillips et al., 2019), they did not significantly differ from WTs (Figure 3A). However, *Wac* Hets did show significant impairments in the Y-maze that tests working memory (Kraeuter et al., 2019a; Lalonde, 2002; Figure 3B, *p*=0.047). Anxiety (elevated plus maze) and spatial memory (radial arm water maze) were not grossly changed but similar to the social discrimination test, cohorts of *Wac* Hets trended towards impairment in each test (Figure 3—figure supplement 1).

**Figure 3—figure supplement 1.**
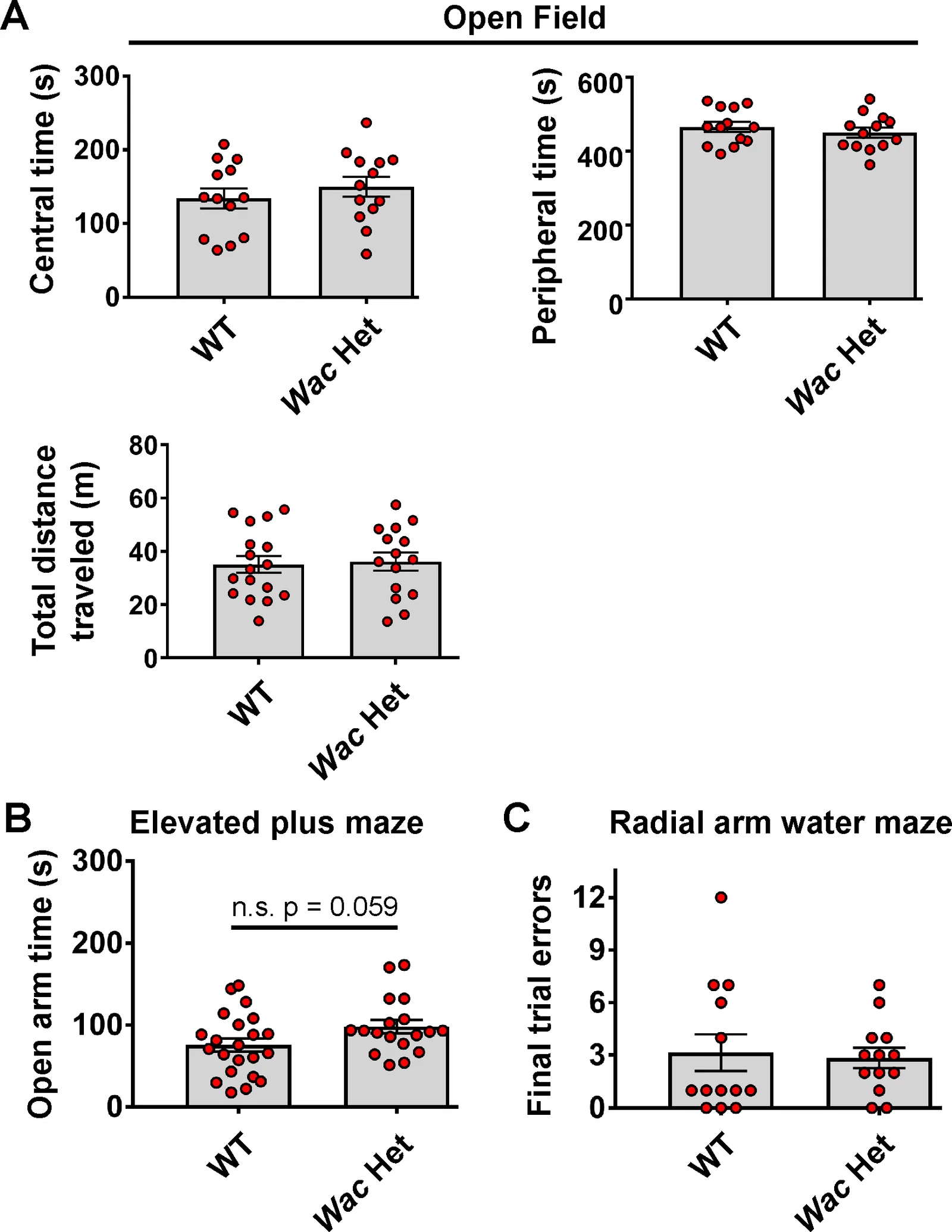
Ambulatory, spatial memory and anxiety-relevant tests in *Wac* Het mice. Adult wild-type (WT) and *Wac* Het mice were assessed for ambulatory activity in the open field test (**A**); no changes were found in time spent in the center (top left graph) or periphery (top right graph), as well as total distance travelled (bottom left graph) between groups. (**B**) The elevated plus maze was used to determine whether mice preferred a safe enclosed arm or open arms. While not significant, *Wac* Het mice trended towards preference for the open arms. (**C**) A spatial memory test was used to test if *Wac* Hets had deficits in spatial memory but as a group no significant differences were observed. Data are expressed as the mean ± SEM, n=13 for both groups for open field periphery measurements, n=17 (WT) and 15 (Het) for total distance. For the radial arm water maze, n=22 WTs and 18 Hets for the elevated plus maze.

We next tested zebrafish in a social cohesion assay; WT fish stayed in close contact while *waca* KOs were dispersed during the trial, see heatmap distribution of zebrafish in Figure 3C and D. While both male and female zebrafish *waca* KOs showed deficits in this assay, we report the sexes independently since WT males and females had significant differences in baseline social cohesion. Moreover, male zebrafish *waca* KOs had a greater change in social cohesion than females (Figure 3E and F, *p*=0.0007 males, *p*=0.01 females). In a novel tank assay to test hyperactivity, male *waca* KO fish moved greater distances throughout the test compared to WTs (Figure 3G, *p*=0.005), while there were no significant difference in females (Figure 3H). While differences exist in zebrafish locomotion/hyperactivity, both models share deficits in some behaviors.

### Elevated seizure susceptibility and depleted GABAergic markers in *Wac* heterozygous mice

The previous behavioral changes suggested that core neuron cell ratios might be altered, potentially through an imbalance in GABAergic/glutamatergic neurons that could impact this and other disorders (Rubenstein and Merzenich, 2003; Sohal and Rubenstein, 2019). Epilepsy occurs frequently in individuals with DESSH syndrome (Alawadhi et al., 2021; Alsahlawi et al., 2020; DeSanto et al., 2015; Leonardi et al., 2020; Lugtenberg et al., 2016), thus, we first tested if loss of *Wac* expression was sufficient to increase seizures in our mouse model. We assessed whether the GABAA receptor antagonist, pentylenetetrazol (PTZ), could induce seizures in mice with *Wac* loss of function. The drug was administered intraperitoneal at a subthreshold dose (50 mg PTZ per kilogram of mouse body weight) that rarely elicited a tonic-clonic seizure in WTs. We hypothesized that if loss of *Wac* promoted a shift favoring brain excitation/inhibition, then *Wac* hets would exhibit more severe seizure behaviors by P30.

**Figure 4.**
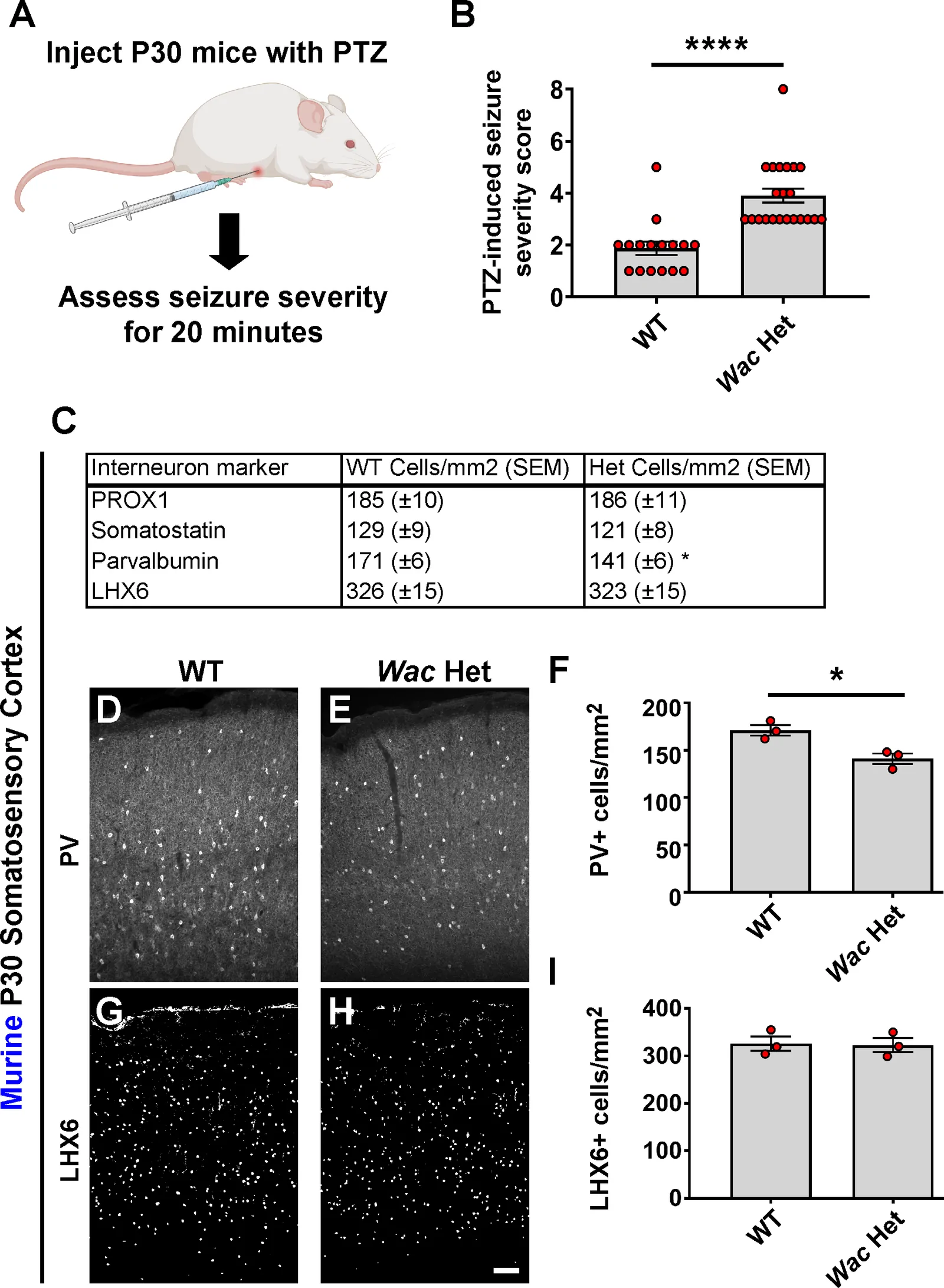
Murine *Wac* depletion leads to elevated seizure susceptibility and loss of GABAergic markers. (**A**) Schema depicting the seizure induction by pentylenetetrazol (PTZ); mouse image was made by BioRender software. Briefly, P30 mice were administered PTZ intraperitoneally and then assessed for the highest seizure severity score over the course of 20 min. The maximum seizure severity score over 20 min was quantified (**B**); n=16 (wild-types, WTs) and 22 (Hets). (**C**) Table of cortical interneuron cell counts in the somatosensory cortex at P30. (**D–F**) Immunofluorescent images of parvalbumin (PV) and cell density quantification in mice somatosensory cortex, n=3, both groups. (**G–I**) Immunofluorescent images of LHX6 and cell density quantification in mice somatosensory cortex, n=3, both groups. Data are expressed as the mean ± SEM, *p<0.05 and ****p<0.0001. Scale bar = 100 µm (**H**).

PTZ was administered and mice were observed for 20 min (Figure 4A); most mice fully recovered by 15 min and all WT mice did not exhibit any behavioral changes by 20 min post-injection. Behaviors included freezing, motor twitches, loss of balance, seizures and hyperactivity, which are detailed in the methods. While PTZ led to subtle behavioral changes in WT mice but rarely elicited a seizure. However, *Wac* Hets exhibited elevated seizure behaviors, including hunched body posture, arching tail, and forelimb clonus, as well as succumbing to seizures (Figure 4B, *p*<0.0001). Thus, loss of *Wac* leads to increased seizure susceptibility in mice, consistent with one of the comorbidities observed in ~40% of individuals with DESSH syndrome. In contrast, when zebrafish were tested for seizure susceptibility, the *waca* KOs did not show any gross changes although both WTs and mutants responded to PTZ (data not shown), suggesting that this specific symptom was not conserved between the 2 vertebrate models.

**Figure 4—figure supplement 1.**
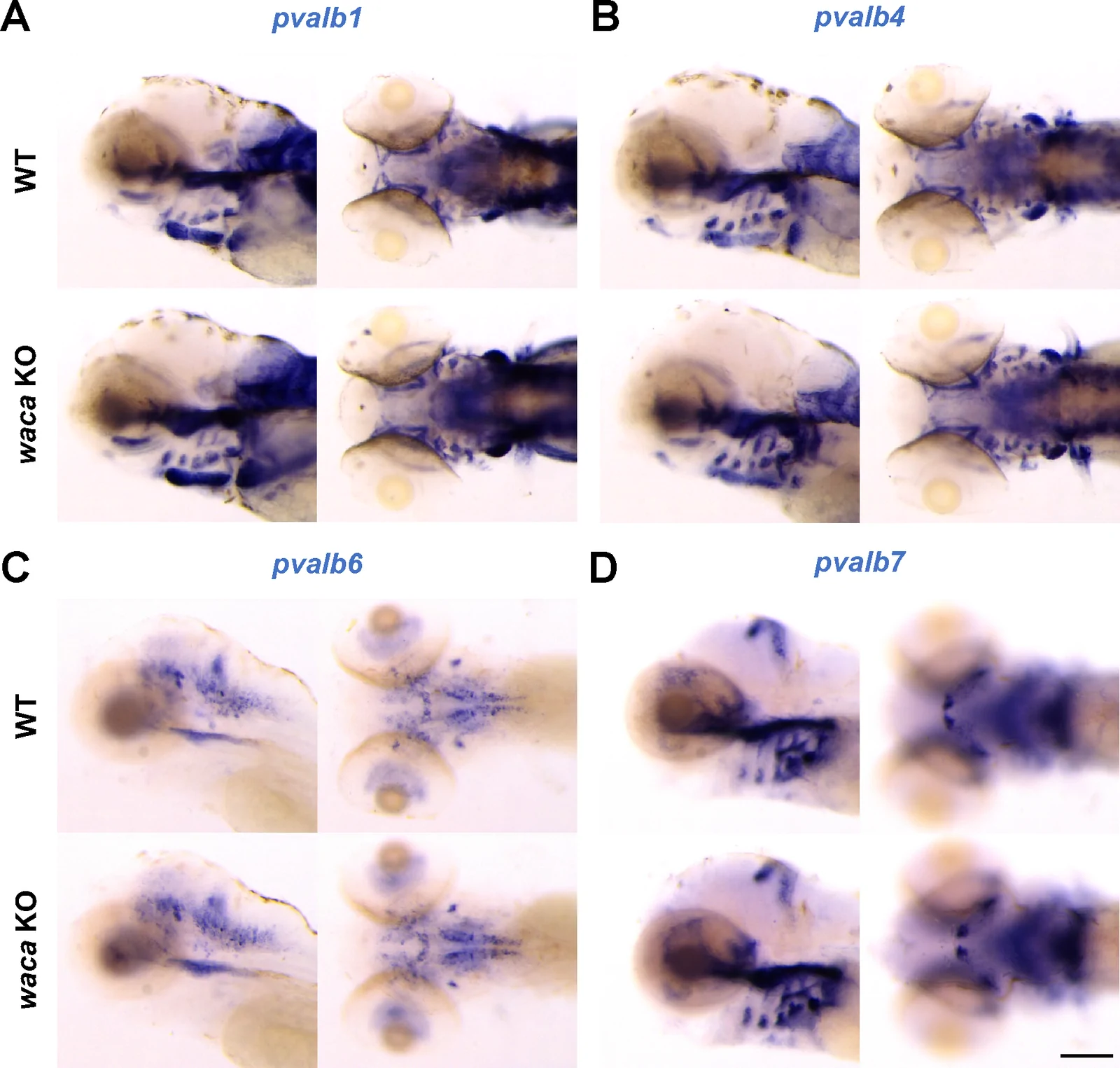
Expression pattern of parvalbumin genes (*pvalb1*, *pvalb4*, *pvalb6*, and *pvalb7*) in *waca* KO zebrafish at 7 dpf. (**A–D**) Lateral view in left panels; dorsal view in right panels; anterior is to the left. No significant change was detected between wild-type (WT) and *waca* KO. n=7 for WT and n=6 for *waca* KO for *pvalb1*. n=5 for WT and n=9 for *waca* KO for *pvalb4*. n=4 for WT and n=6 for *waca* KO for *pvalb6*. n=7 for WT and n=3 for *waca* KO for *pvalb7*. Scale bar in (**D**) = 200 µm.

**Figure 4—figure supplement 2.**
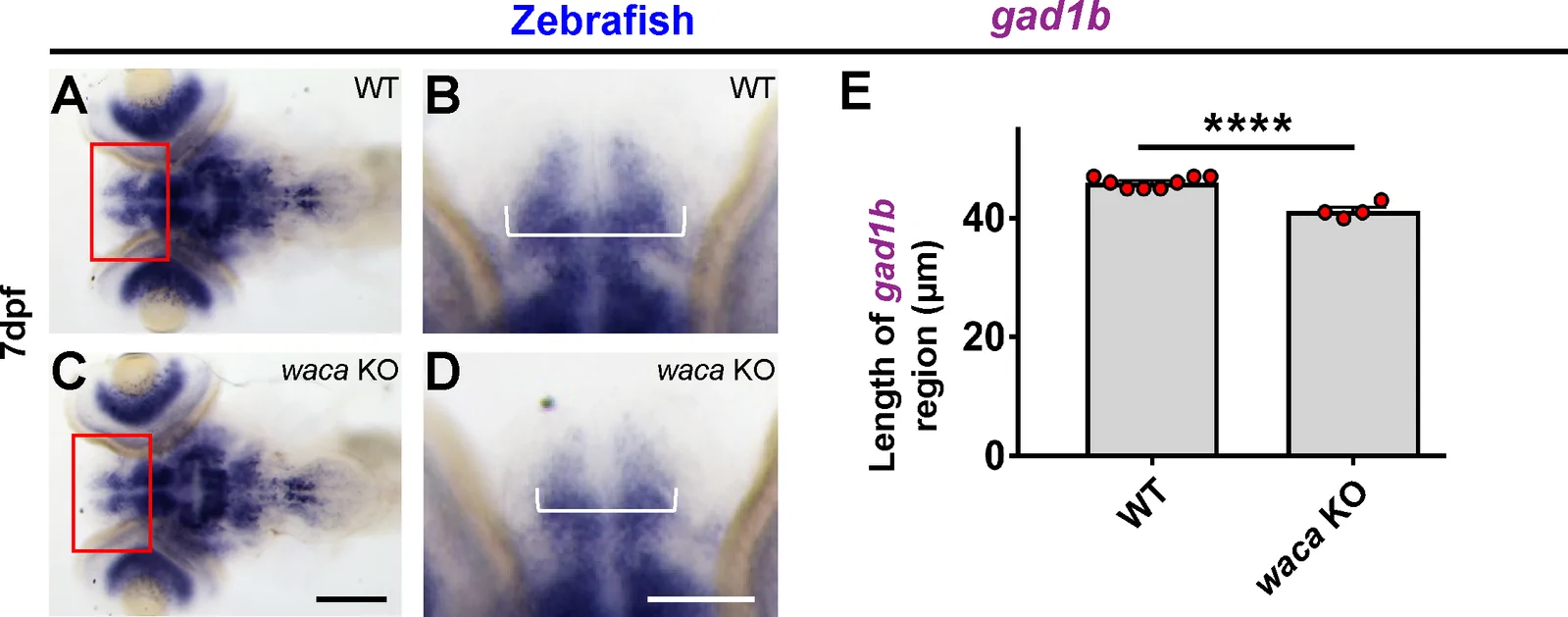
Expression pattern of the *gad1b* gene in *waca* KO zebrafish at 7 dpf. (**A–D**) Dorsal view of *gad1b* labeling, with anterior to the left (in left panels) and to the top (in right panels). Boxes highlight forebrain regions. Brackets demarcate area of *gad1b* expression. (**E**) Quantification of the *gad1b* area of expression between wild-type (WT) and *waca* KOs. Data are expressed as the mean ± SEM, n=8 for WT and n=4 for *waca* KO, ****p<0.0001. Scale bars in (**C**) = 200 µm, (**D**) = 200 µm.

### Some GABAergic deficits in mouse and zebrafish *Wac/wac* mutants

While we only found a susceptibility to seizures in mice, an imbalance in excitatory and inhibitory cell populations may still exist in each model but with differential impacts on seizures and/or behaviors. One possibility is a decrease in GABAergic cortical interneuron (CIN) function, a diverse group of inhibitory neurons whose dysfunction has been implicated in other syndromes with epilepsy and/or autism (Angara et al., 2020; Kim et al., 2018; Malik et al., 2019; Vogt et al., 2018; Vogt et al., 2015; Vogt et al., 2014). To assess whether *Wac* may impact CIN populations, we assessed three broad groups of CINs; parvalbumin (PV) + and somatostatin (SST)+, which label ~70% of GABAergic interneurons (Wonders and Anderson, 2006), and prospero homeobox 1 (PROX1) expressing, which labels the majority of caudal-derived interneurons (Rubin and Kessaris, 2013). The cell density of SST and PROX1 CINs was normal, but there was ~18% decrease in PV + CINs in the Het (Figure 4C–F, *p*=0.02); example images of SST and PROX1 cells are shown (Figure 5—figure supplement 1). To probe whether PV expression is due to loss of cells or simply the expression of PV, we assessed for the CIN marker, LHX6, which is expressed in all PV + CINs in the somatosensory cortex (Angara et al., 2020). We found no decrease in LHX6 cell numbers (Figure 4C and G–I), suggesting this phenotype is likely due to impacts upon PV expression rather than loss of this group of interneurons.

We also studied GABAergic markers in *waca* KO zebrafish. We first probed via in situ for the various parvalbumin transcripts in zebrafish but found no differences, including *pvalb6*, which is the only one expressed in the brain (Figure 4—figure supplement 1). We next looked at the pan-GABAergic gene, *gad1b*, in the forebrain and compared *gad1b* expression in WTs and *waca* KOs. Notably, we uncovered a significant reduction in forebrain area that expressed the transcript (Figure 4—figure supplement 2, *p*<0.0001); WT and *waca* KO zebrafish brains were not altered in general size, suggesting this measurement was not biased due to a smaller or larger brain due to genotype. These and previous data suggest that GABAergic neuron populations are impacted by mouse and zebrafish *Wac*/*waca* loss-of-function and potentially contribute to DESSH syndrome.

### Normal numbers of major brain cell types and proteins in *Wac* mutant mice brains

**Figure 5.**
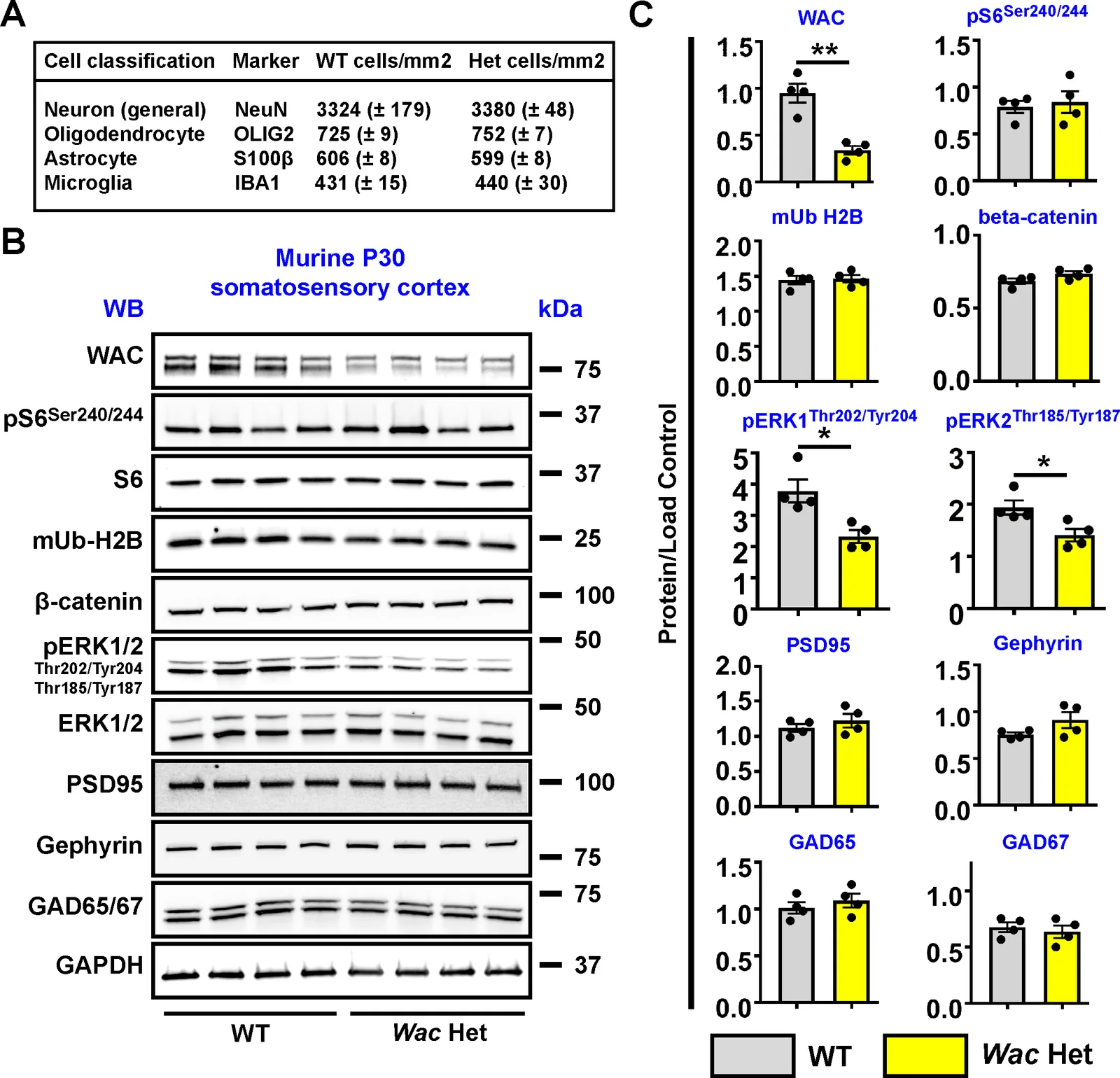
Cell counts and biochemical assessment of key markers in P30 *Wac* wild-type (WT) and Het mice. (**A**) WT and *Wac* Het somatosensory cortices were counted for major cell classes, including markers of neurons and glia, n=3 each group, parentheses indicate standard error of the mean. (**B**) P30 somatosensory cortex tissue was probed via western blotting for WAC and proteins known to be altered in other models or of brain markers. (**C**) Quantification of protein bands were normalized to GAPDH or the total protein if a phosphorylated protein. Data are expressed as the mean ± SEM, n=4 all groups. *p<0.05 and **p<0.01. Abbreviations: (WB) Western blot and (kDa) kiloDaltons. Figure 5—source data 1.Compiled western blots. Figure 5—source data 2.Wac western blot. Figure 5—source data 3.beta-catenin western blot. Figure 5—source data 4.ERK western blot. Figure 5—source data 5.GAD65/67 western blot. Figure 5—source data 6.GAPDH western blot. Figure 5—source data 7.Gephyrin western blot. Figure 5—source data 8.mono-ubiquitin H2B western blot. Figure 5—source data 9.phospho-ERK western blot. Figure 5—source data 10.phospho-S6 western blot. Figure 5—source data 11.PSD95 western blot. Figure 5—source data 12.S6 western blot.

Since many antibodies are not available for zebrafish, and mice are closer to humans in gene conservation, we chose to focus on our mouse model to next assess if any other major brain cell populations, signaling events (including post-translational modifications), or gross synaptic markers were altered by loss of *Wac*. Thus, we first performed cell density counts for different classes of neuronal cells in our mouse model that included neurons, oligodendrocytes, astrocytes, and microglia in P30 somatosensory cortices (Figure 5A). The total number of neurons was assessed by labeling for NeuN, while oligodendrocytes, astrocytes and microglia were labeled with OLIG2, S100beta and IBA1, respectively; representative images of these markers are shown in Figure 5—figure supplement 1. No differences in these major cell types were observed between the genotypes in mice.

**Figure 5—figure supplement 1.**
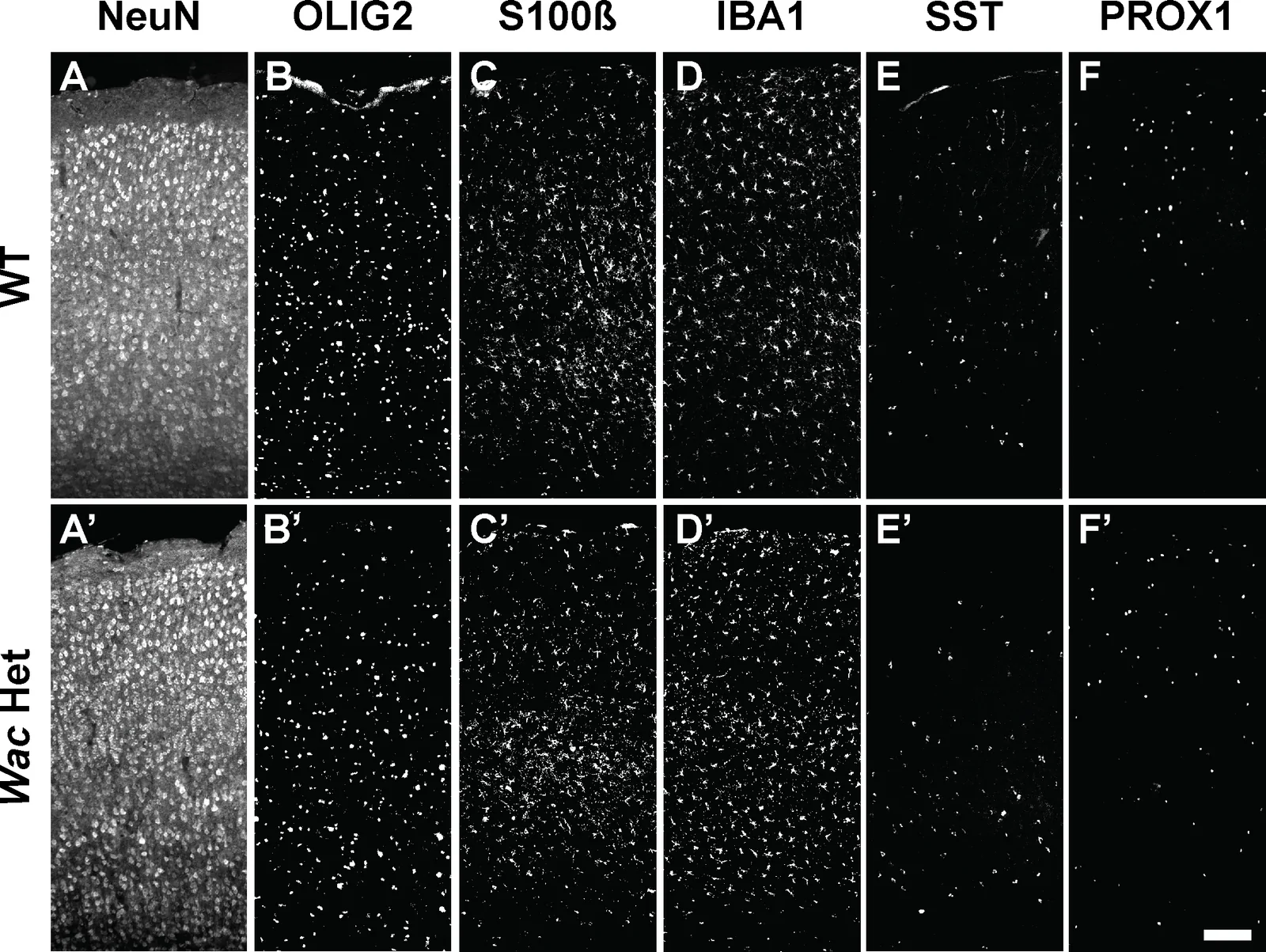
Example images of cortical glial and neuron cell types in wild-type (WT) and *Wac* Het brains. Scale bar in F’=100 µm.

**Figure 5—figure supplement 2.**
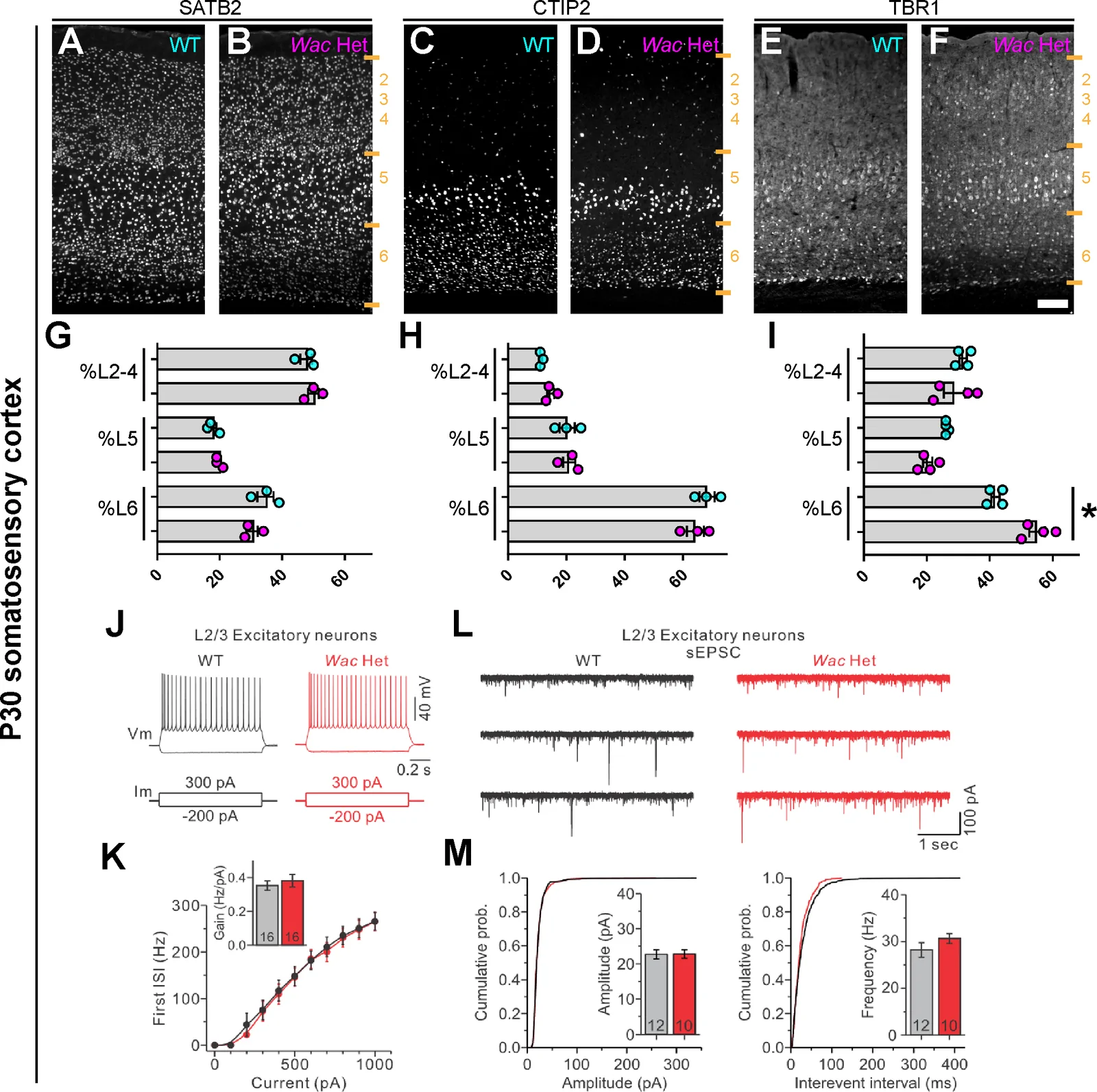
Normal pyramidal neuron lamination, intrinsic membrane properties, and spontaneous excitatory postsynaptic currents in the somatosensory cortex. Representative images of pyramidal neuron laminar markers in somatosensory cortices of P30 wild-type (WT) and *Wac* Hets for SATB2 (**A, B**), CTIP2 (**C, D**) and TBR1 (**E, F**); scale bar in F=100 µm. Quantification of the % marker per layer for SATB2 (**G**) n=3, CTIP2 (**H**) n=3 and TBR1 (**I**) n=4. (**J**) Example voltage responses of wild-type (WT) and *Wac* Het L2/3 excitatory neurons to injected current. (**K**) Frequency-intensity (FI) plots showing the first interspike interval (ISI) frequency for WT and *Wac* Het L2/3 excitatory neurons following positive current steps (0–1000 pA; 100 pA steps; 1000 ms duration) (WT: 16 cells from 3 mice; *Wac* Het: 16 cells from 3 mice). Bar graphs show that the mean linear slopes (gain) of the FI curves were not significantly different (WT: 0.35±0.03 Hz/pA; *Wac* Het: 0.38±0.04 Hz/pA; *p*=0.54, two-sample t-test). (**L**) Three representative traces of spontaneous excitatory postsynaptic currents (sEPSCs: 5 s per trace) recorded in voltage-clamp (–94 mV) from a WT and *Wac* Het L2/3 excitatory neuron. (**M**) Cumulative probability plots of sEPSC amplitudes (left) and interevent intervals (right) from WT and *Wac* Het L2/3 excitatory neurons (WT: 540 events, 12 cells from 3 mice; *Wac* Het: 450 events, 10 cells from 3 mice). Bar graphs show no significant differences in the mean sEPSC amplitude (left) or frequency when averaged from individual L2/3 excitatory neurons (WT amplitude: 22.7±1.3 pA; *Wac* Het amplitude: 22.8±1.2 pA, *P*=0.94, two-sample t-test) (WT frequency: 36.4±3.1 Hz; *Wac* Het: 41.3±2.1 Hz, *p*=0.23, two-sample t-test). Values are represented as mean ± SEM, *p<0.05.

Next, we assessed mouse P30 somatosensory cortices for proteins changed in other *Wac* models or markers of brain synapses. As mentioned earlier, WAC protein was reduced 64% in the Hets (Figure 5B and C, *p*=0.002). Despite previous reports in other models (David-Morrison et al., 2016; Zhang and Yu, 2011), we did not observe differences in mTOR activity or ubiquitination of histone 2B. Key pathways, including Wnt/beta-catenin and synaptic markers, PSD95 (excitatory synapses) and Gephyrin/Gad65/67 (inhibitory synapses), were not altered (Figure 5B and C). For the MAPK signaling pathway, there was a decrease in active ERK1, 38% and ERK2, 33%, in the *Wac* Hets (Figure 5B and C, pERK1 *p*=0.01, pERK2 *p*=0.02), suggesting that while major cell types and proteins involved in brain function are not altered, some signaling events may be candidates for future studies to understand WAC function.

### Subtle pyramidal neuron lamination but normal glutamatergic electrophysiological properties in mouse *Wac* Hets

Our data thus far revealed altered GABAergic neuron marker expression but no gross changes in other generic neuron numbers, glia numbers, or synaptic protein abundance. However, these observations could still be true while subtypes of excitatory neurons in distinct cortical lamina or their physiological/synaptic properties are yet to be altered. Thus, we also assessed glutamatergic neuron laminar markers in the somatosensory cortices of P30 mice. SATB2, CTIP2 and TBR1 were labeled and the proportion of expressing neurons assessed in layers 2/3/4, 5, and 6 at P30. While there were no significant laminar changes in the Het for SATB2 and CTIP2, there was a proportional increase of TBR1 + cells in layer 6 (Figure 5—figure supplement 2, *p*=0.047). We also examined the intrinsic membrane properties of layer 2/3 neurons in the primary somatosensory cortex of adult WT and Het mice. Overall, there were no gross differences in passive or active membrane properties between *Wac* Het and WT cells (Figure 5—figure supplement 2 and Table 1), suggesting excitatory cortical neurons in the mutants have normal physiological properties. We also assessed synaptic function by measuring spontaneous excitatory postsynaptic currents (EPSCs) in layer 2/3 neurons. We found no differences between WT and *Wac* Het excitatory neurons (Figure 5—figure supplement 2), suggesting that local excitatory neurons in the neocortex are not measurably impacted by *Wac* depletion in the Het mice.

**Table 1.** Electrophysiological properties of L2/3 excitatory cells in the somatosensory cortex of wild-type (WT) and *Wac* Het mice.

	L2/3 excitatory neurons						*P*	Test
	WT			*Wac* Het				
	Mean±s.e.m.			Mean±s.e.m.				
RMP (mV)	–86.8	±	0.7	–87.1	±	0.6	0.747	*t-*test
R_m_ (MΩ)	66.8	±	3.6	59.2	±	3.7	0.151	*t-*test
τ_m_ (ms)	12.9	±	0.5	12.3	±	0.4	0.209	*t-*test
C_m_ (pF)	197.4	±	8.8	218.3	±	13.7	0.720	MW *U*-test
Rheobase (pA)	170.0	±	11.0	167.2	±	12.6	0.868	*t-*test
Spike threshold (mV)	–49.9	±	0.6	–50.1	±	0.5	0.788	*t-*test
Spike amplitude (mV)	85.0	±	1.5	88.1	±	1.4	0.142	*t-*test
Spike half-width (mV)	0.63	±	0.02	0.61	±	0.02	0.323	*t-*test
AHP amplitude (mV)	–18.3	±	0.6	–18.5	±	0.5	0.794	*t-*test
Max rate of rise (mV*ms)	526.0	±	28.8	586.3	±	22.4	0.109	*t-*test
Max rate of decay (mV*ms)	–112.8	±	4.2	–120.0	±	4.5	0.243	*t-*test
	n=16 cells, 3 mice			n=16 cells, 3 mice				

^*^See methods for an explanation of how the electrophysiological parameters were defined/measured. All membrane potentials were corrected for a 14 mV liquid junction potential. All statistical tests were two-tailed.

### Increased brain volume in *Wac* Het mice with a male bias

**Figure 6.**
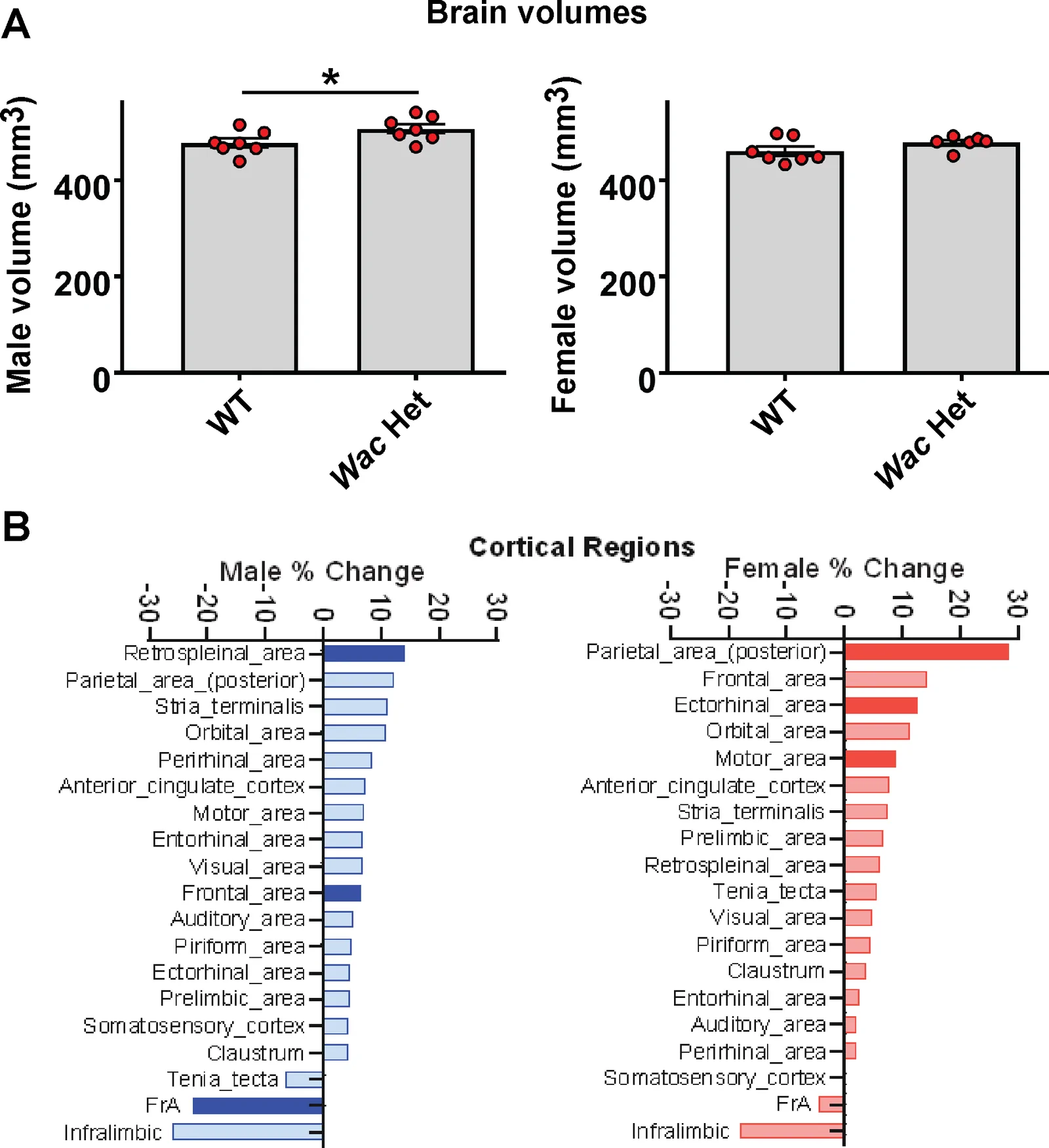
Magnetic resonance imaging (MRI) reveals increased brain volume in mouse *Wac* Het males. (**A**) There was a significant increase in whole brain volumes in HET compared to wild-type (WT) male mice. Female HET mice did not exhibit significant changes in brain volume relative to WT. Data are expressed as the mean ± SEM; males and female WTs n=7 and female Hets n=6, *p<0.05. (**B**) %change between cortical regions were larger in most HET mice areas, with distinct regions different between sexes. (Bolded bars indicate significant differences *p*<0.05, t-test).

While our zebrafish model is similar to the mouse in many core symptoms that present in humans, the *Wac* Het mice recapitulate almost all DESSH syndromes yet tested. Thus, we wanted to take advantage of this model to understand if there may be more changes to the mouse Het brain that could underlie core and other symptoms relevant to humans. Since craniofacial symptoms exist in nearly all DESSH patients and our models have these same alterations, we hypothesized that alterations to the skull shape may also be correlated with altered brain volume. To determine what regions of the mutant mouse brains may be anatomically different, we performed magnetic resonance imaging (MRI) on P30 fixed WT and *Wac* Het brains of both sexes. We first assessed total brain volume of the WT and Hets and found that male *Wac* mutants exhibit a larger whole brain volume than WT males; there were no significant differences in female brain volumes (Figure 6A, *p*=0.04). Next, we examined specific regional volumetric changes, starting with cortical domains. Several alterations were observed in both males and some in females, with most cortical regions showing an increase in volume compared to WTs. Notably, only males had increased retrosplenial cortices, while females showed increases in parietal cortices (Figure 6B, retrosplenial cortex male Het vs male WT *p*=0.002 and female WT vs. female Het *p*=0.01).

**Figure 6—figure supplement 1.**
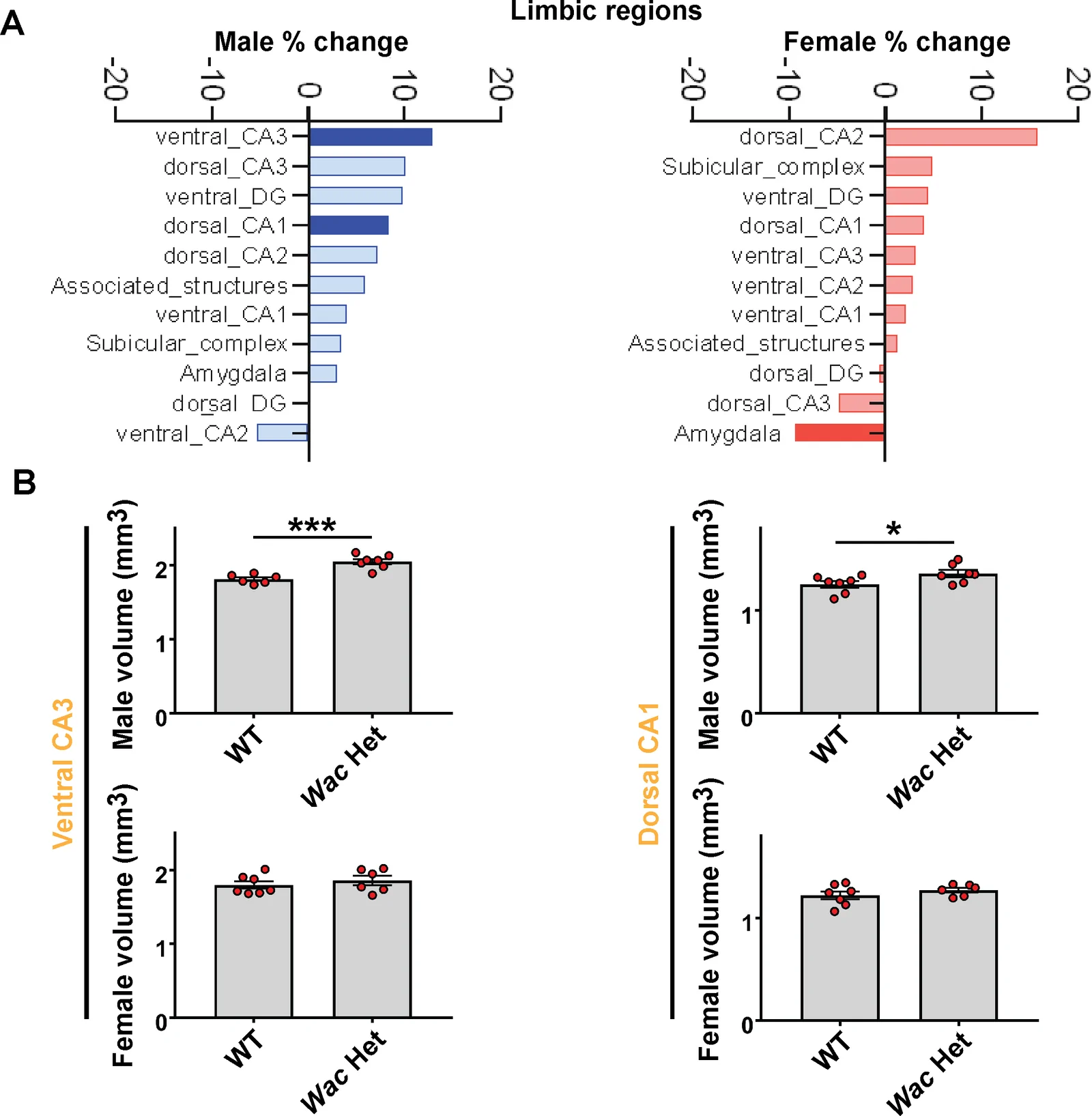
Volumetric limbic regional changes. (**A**) Percent change in limbic regions were modestly larger in HET mice than wild-type (WT). In males, the ventral CA3 and dorsal CA1 were larger but not in females. Female HET mice had smaller amygdala than WT female mice. (Bolded bars indicate significant differences *P*<0.05, t-test). (**B**) Ventral CA3 was significantly larger in HET male mice but not in female mice. Similarly, the dorsal CA1 also exhibited increased volumes in males HET mice but not WT nor in females. Data are expressed as the mean ± SEM; all groups n=6–7, (*p<0.05; ***p<0.001).

**Figure 6—figure supplement 2.**
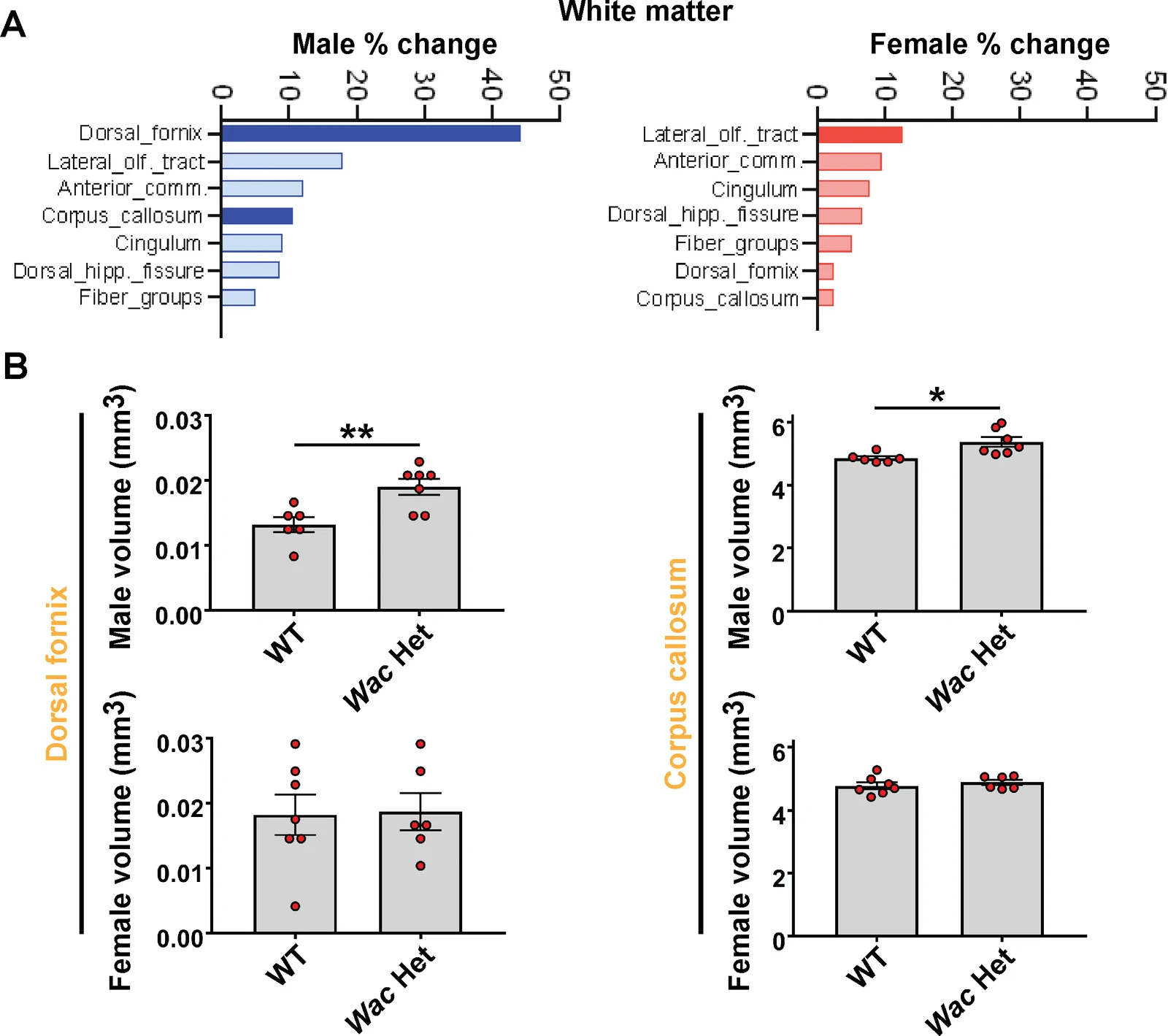
Volumetric changes in white matter. (**A**) Percent change in white matter regions were larger in male HET mice compared to wild-type (WT), particularly the dorsal fornix. In females, only the lateral olfactory tract was larger in HET mice than WT. (Bolded bars indicate significant differences *p*<0.05, t-test). (**B**) There was an increase in dorsal fornix volume in male but not female HET mice compared to WT. There was a robust increase in corpus callosum volume in HET male mice compared to WT but not in female HET mice. Data are expressed as the mean ± SEM; all groups n=6–7, (*p<0.05; **p<0.01).

We also examined limbic regions and white matter tract volumes in WT and *Wac* Het mice. As before, male mice had significant increases in both limbic and white matter volumes compared to females. Specifically, hippocampal ventral CA3 and dorsal CA1 regions were increased in males but not females (Figure 6—figure supplement 1, ventral CA3 male WT vs. male Het *p*=0.0003 and dorsal CA1 male WT vs. male Het *p*=0.04). Male-specific increased volume was also observed in white matter dorsal fornix and corpus callosum tracts (Figure 6—figure supplement 2, dorsal fornix male WT vs. male Het *p*=0.006 and corpus callosum male WT vs. male Het *p*=0.01). As of this report, no DESSH syndrome individuals have been MRI assessed. However, these observations may be potential predictors of phenotypes that may arise as those with DESSH syndrome age into adulthood and whether there could be sex differences in humans.

### RNA sequencing of mouse forebrains reveals DESSH syndrome candidate transcripts

In a similar logic to our above tests, we chose to focus on the mouse model to assess potential RNA transcripts that are changed due to *Wac* loss-of-function since the mouse model may be more relevant to humans with DESSH. To discover potential molecular changes that could inform future mechanistic studies, we compared WT and *Wac* Het bulk RNA-seq at postnatal day 2 (P2), an age when mice are undergoing critical developmental milestones, including neuronal migration, cell death, axon and dendrite outgrowth, as well as beginning synaptogenesis. We sequenced 14 WT and 10 *Wac* Het sex-balanced forebrain samples to a median sequencing depth of 50 million paired-end reads per sample (Figure 7—figure supplement 1; Supplementary file 1). As expected, we found significant correlations between sample sex and sequencing depth, and principal component analysis (PCA) dimensions; no outliers were detected in the PCA space (Figure 7—figure supplement 2; Supplementary file 1).

**Figure 7.**
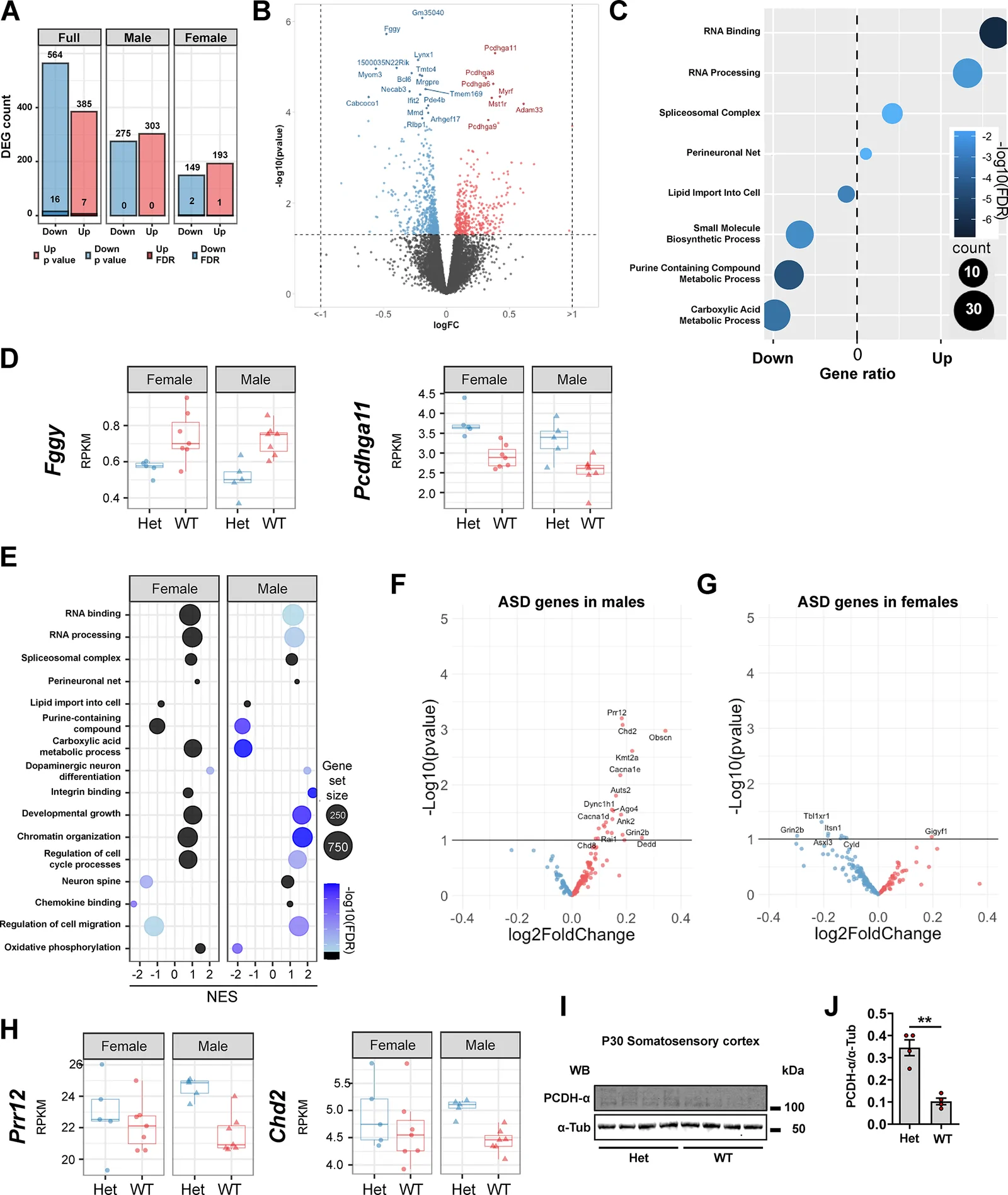
Transcriptomic characterization by bulk RNA-seq identified shared and divergent transcriptomic dysregulation in males and females. (**A**) Number of differential expression (DE) genes identified in the surrogate variable analysis (SVA)-corrected DE analysis and sex-stratified DE analysis. Results are separated by effect direction and significance (*p*-value <0.05 or FDR <0.1). (**B**) Volcano plot representing DE of the SVA-corrected DE model in (**A**). (**C**) Selected GO analysis of significantly (*p*<0.05) up and down-regulated genes in the SVA-corrected DE analysis. Results are colored by significance, the size indicated the number of DE genes belonging to that term and are ordered by the ratio of enrichment. (**D**) Examples of significant DE genes identified in the SVA-corrected model *Fggy* (FDR = 0.014) and *Pcdhga11* (FDR = 0.026), stratified by sex. (**E**) Curated GO terms from stratified GSEA analysis. Colors indicate significance, size the number of genes in the enriched term, and the enrichment score (NES). (**F, G**) Volcano plots showing the differential expression of autism spectrum disorder (ASD) genes in males and females. (**H**) Examples of DE genes which show increased effect size and significance in males, *Prr12* (females, *p*=0.99; males, *p*=0.0006) and *Chd2* (females, *p*=0.39; males, *p*=0.0008). (**I**) Western blots showing PCDH-alpha abundance in wild-types (WT)s and Hets. (**J**) WB quantification comparing PCDH-alpha normalized to alpha-tubulin expression in P30 somatosensory cortices; n=4 for both genotypes and **p<0.01. Data are expressed as the mean ± SEM. Figure 7—source data 1.Compiled western blots. Figure 7—source data 2.PCDH alpha western blot. Figure 7—source data 3.alpha tubulin western blot.

Differential expression (DE) analysis was performed using edgeR and included surrogate variable analysis (SVA) batch correction (Leek et al., 2012), which accounted for sex- and batch-biased transcriptomic variability. We found 7 and 16 genes were significantly upregulated and downregulated at FDR <0.1, respectively; and 385 and 564 upregulated and downregulated genes were identified at a more inclusive *p*<0.05 threshold (Figure 7A). DE was skewed towards downregulation, which is consistent with *Wac* function in promoting transcription (Zhang and Yu, 2011). While *Wac* gene expression was modestly upregulated in the mutants (log2FC = 0.09, *p*=0.01, FDR = 0.5), coverage of floxed *Wac* exon 5 was reduced by 50% in HET samples (Figure 7—figure supplement 3), consistent with compensatory upregulation. As noted earlier, western blot found decreased WAC protein expression (Figure 5) and no evidence of mutant isoforms, suggesting that mutant *Wac* transcript is unlikely to be stably translated. Sex-stratified analysis identified three genes passing FDR <0.1 (one upregulated and two downregulated) in females, and 0 in males. At *p*<0.05, the number of DE genes was higher in males, with 303 upregulated and 275 downregulated, in contrast to 193 upregulated and 149 downregulated genes in females (Figure 7A, Supplementary files 1–5); a volcano plot showing example up and down regulated genes is shown in Figure 7B.

**Figure 7—figure supplement 1.**
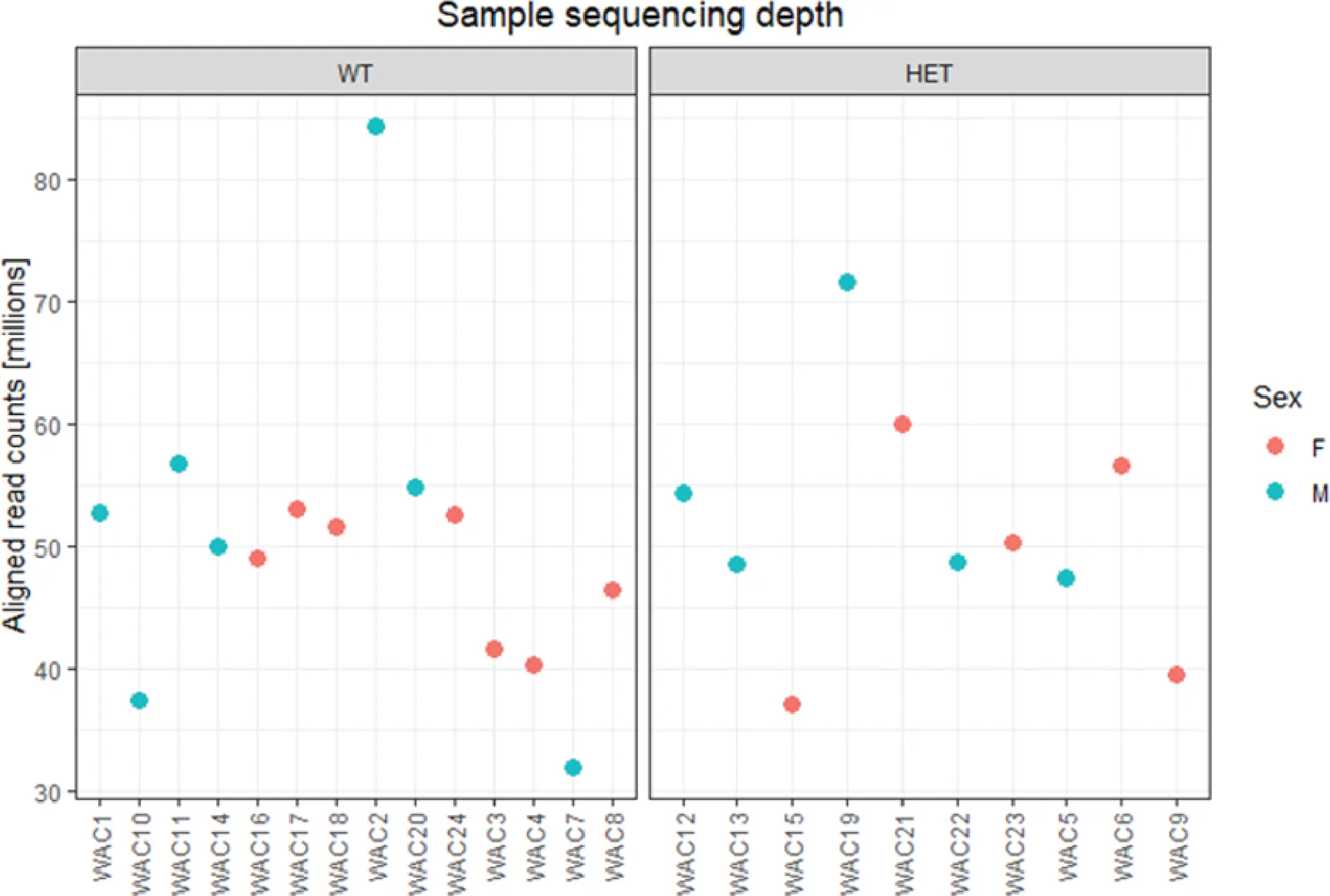
Sample sequencing depth. Values represent millions of aligned reads.

**Figure 7—figure supplement 2.**
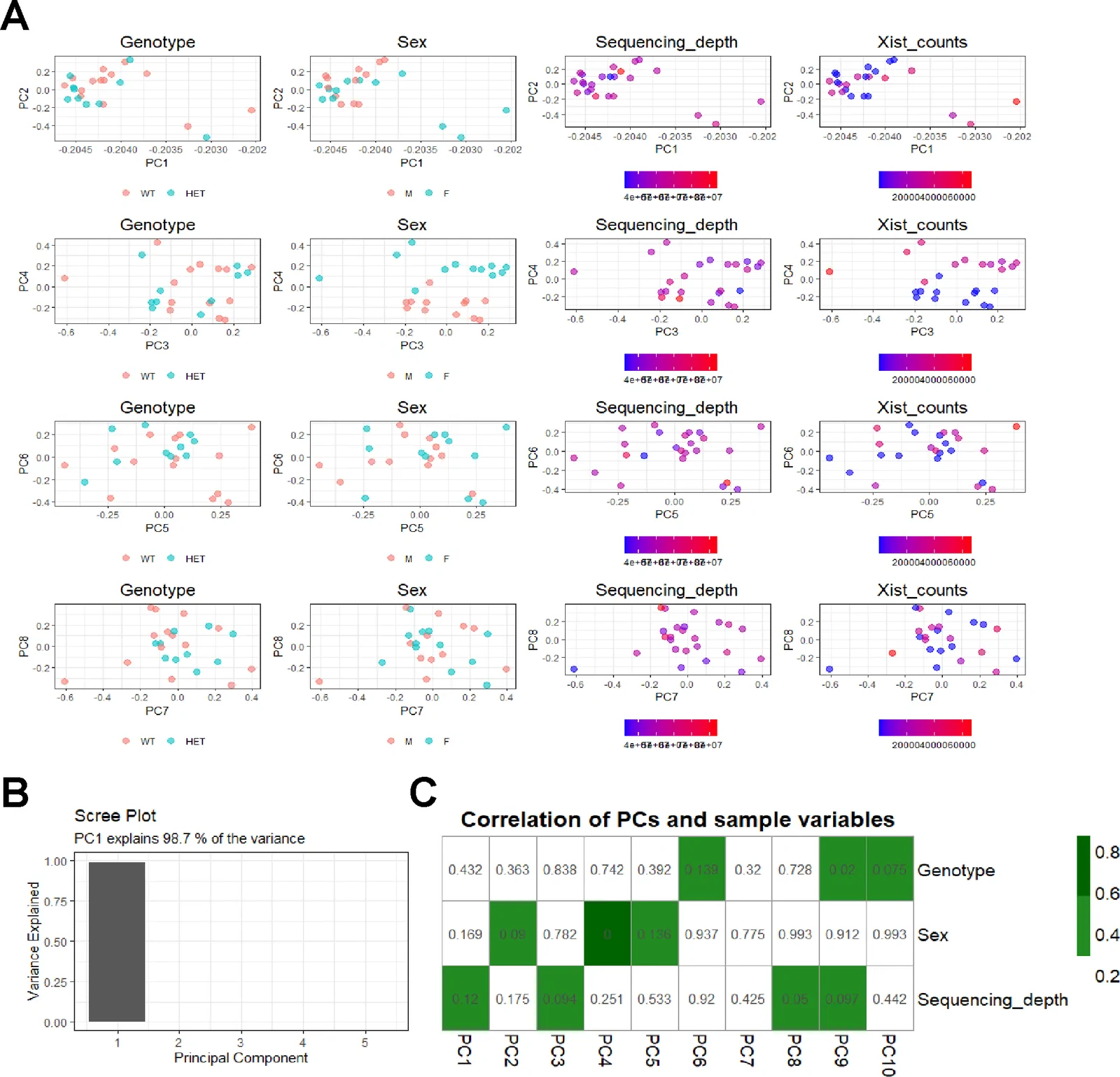
Principal component analysis (PCA) dimensionality reduction. (**A**) PCA sample clustering showing first eight PCs. Samples are colored by genotype, sex, sequencing depth, and Xist gene counts. PCA did not identify sample outliers. (**B**) Scree plot showing the amount of variance explained by the first five PCs. (**C**) Correlation heatmaps of PCs 1–10 vs genotype, sex, and sequencing depth. Values in the heatmap represent correlation *p*-values. The intensity of the heatmap colors represents the Pearson correlation coefficient (**r**).

**Figure 7—figure supplement 3.**
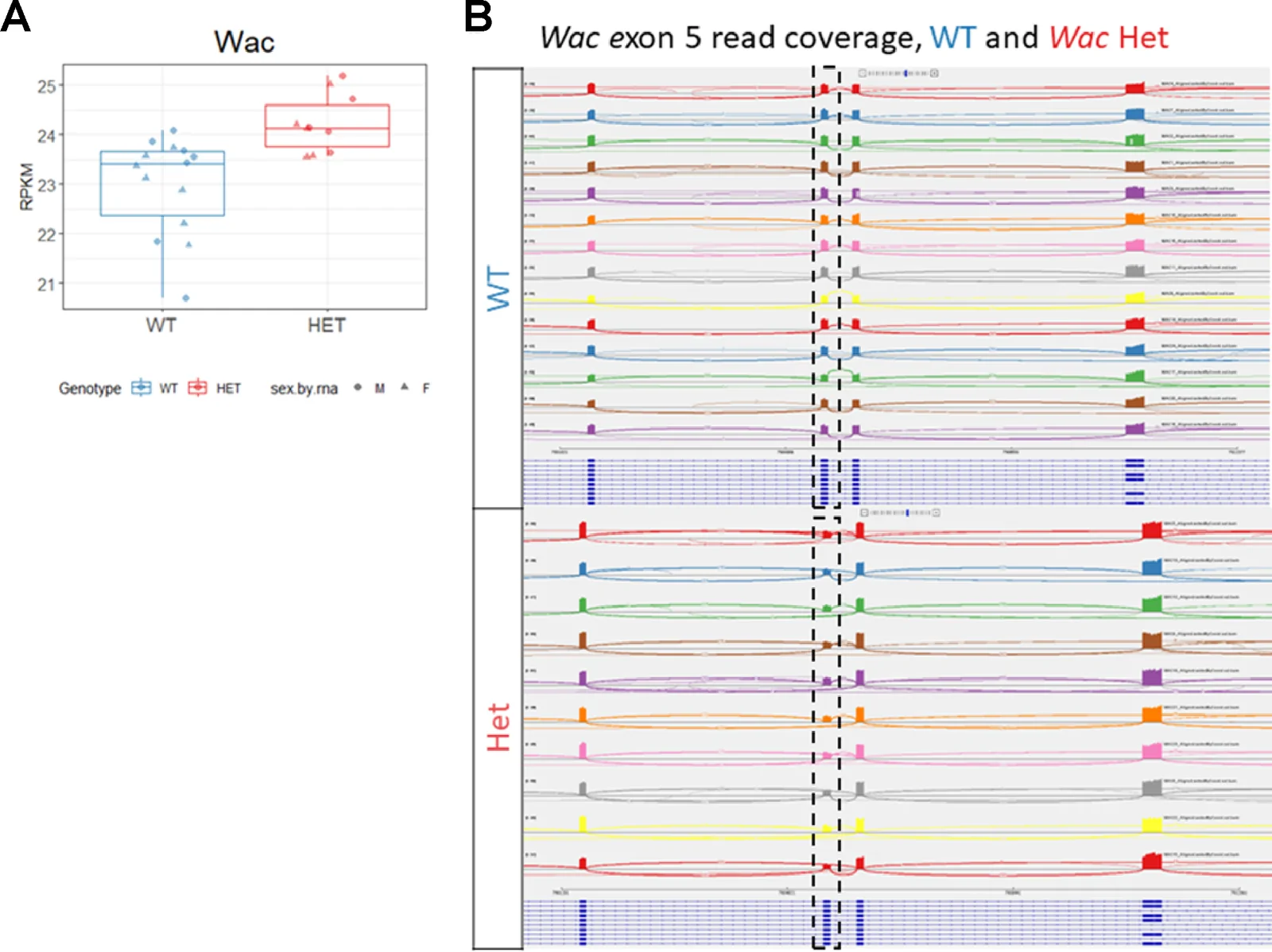
Validation of *Wac* heterozygosity in RNA-seq data. (**A**) *Wac* Het samples demonstrate subtle upregulation of *Wac* transcript, when reads are counted across the entire gene length (log2FC = 0.09, *p*=0.01, FDR = 0.5) suggesting compensatory upregulation of *Wac* transcription. (**B**) Sashimi plots representing *Wac* read coverage. Dashed rectangle marks floxed exon 5, which coverage is reduced by ~50% in Het samples when compared to surrounding exons.

**Figure 7—figure supplement 4.**
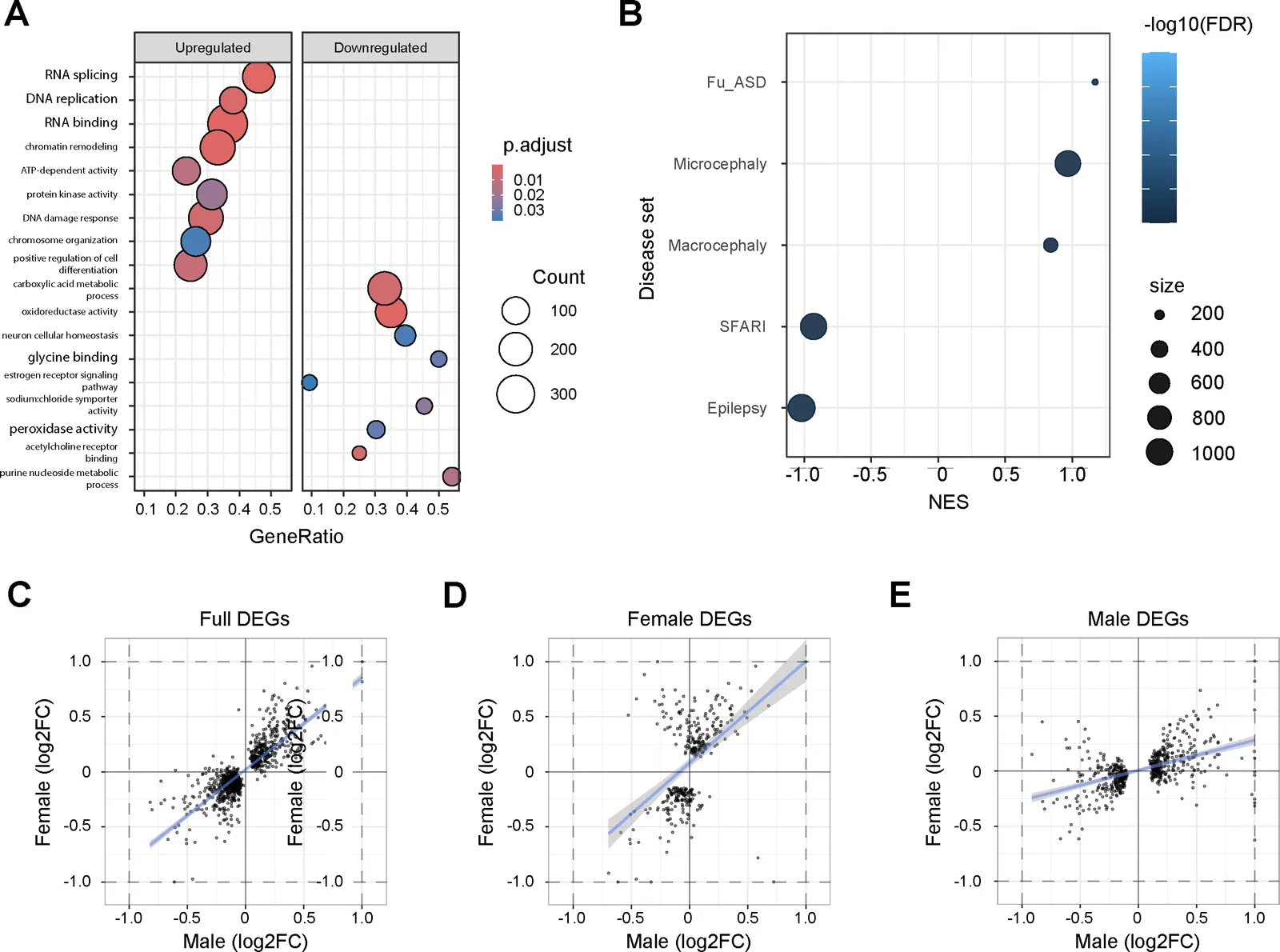
Gene set enrichment and sex concordance in P2 *Wac* mouse forebrain bulk RNA-seq. (**A**) Curated gene ontology (GO) terms from surrogate variable analysis (SVA)-corrected gene set enrichment analysis (GSEA) analysis. Colors indicate significance, size, the number of genes in the enriched term, and the enrichment score (NES). (**B**) Custom GSEA test looking for enrichment of disease-associated genes in the SVA-corrected DE model. Colors indicate significance, size the number of genes in the enriched term, and the enrichment score (NES). (**C**) Correlation of the effected sizes (log fold change) of DEGs (*p*-value <0.05) identified in the SVA-corrected DE analysis between males and females. Line indicated a best-fit linear regression with a 95% confidence interval. (**D**) Correlation of the effected sizes (log fold change) of DE genes (*p*-value <0.05) identified in the female DE analysis between males and females. Line indicated a best-fit linear regression with a 95% confidence interval. (**E**) Correlation of the effected sizes (log fold change) of DE genes (*p*-value <0.05) identified in the male DE analysis between males and females. Line indicated a best-fit linear regression with a 95% confidence interval.

**Figure 7—figure supplement 5.**
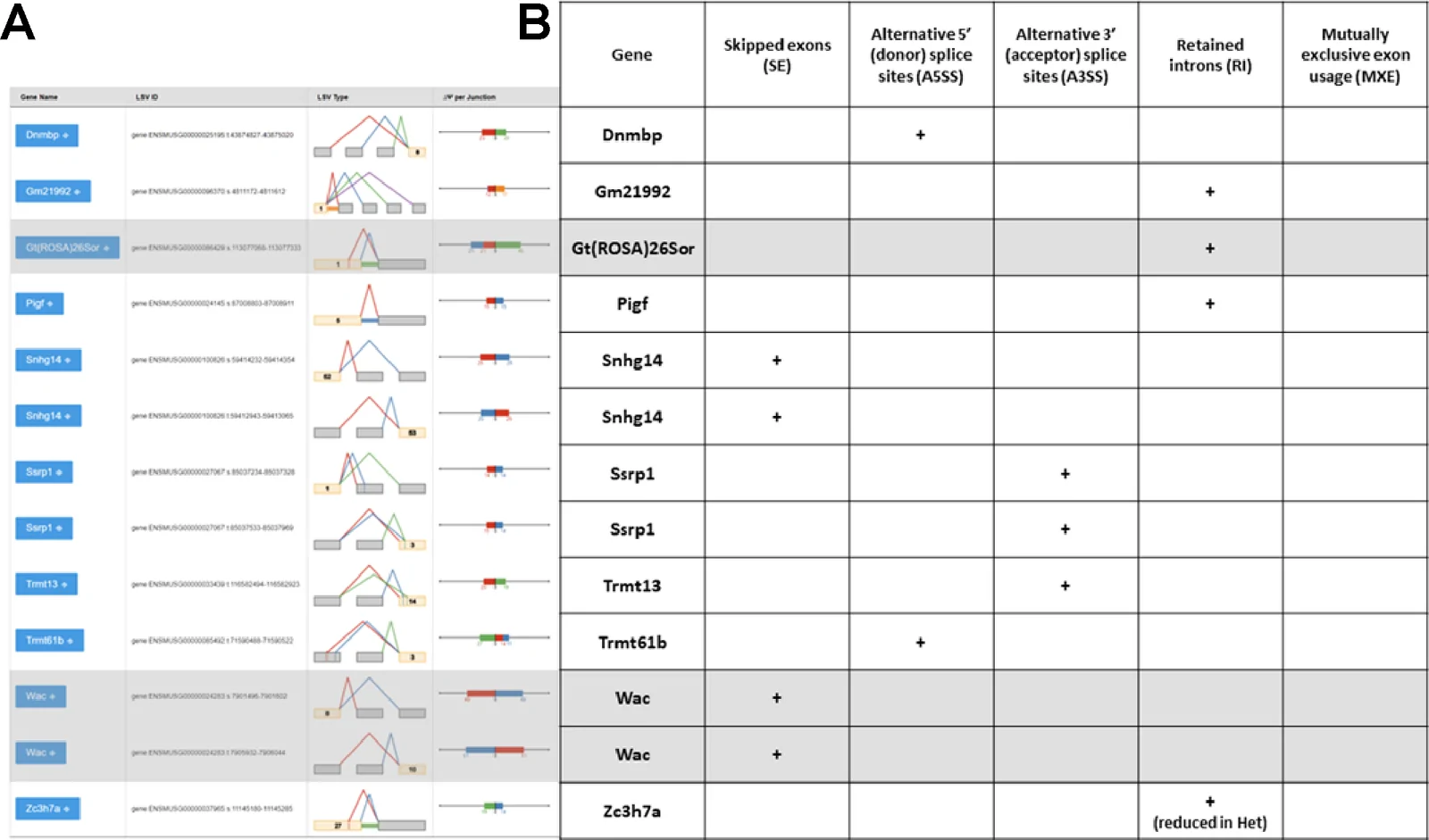
Summary of differential splicing (DS) analysis. (**A**) Visualizations of DS events, including splice graphs. Values in the ΔΨ per junction column represent percentage of the expected differential inclusion for matching edges in the splice graph. (**B**) DS event classification. DS events indicated in gray result from transgenesis.

We performed gene ontology (GO) enrichment analysis of the DE genes passing a *p*<0.05 threshold in the sex-combined model (Figure 7C). Upregulated DE genes were enriched for biological processes involved in RNA processing and splicing, whereas downregulated DEGs were enriched for metabolic processes clustered around purine and fatty acid metabolism. Gene set enrichment analysis (GSEA), which uses a rank-based approach to determine enriched pathways amongst up- and down-regulated genes, also identified RNA processing-related terms to be upregulated and metabolic terms downregulated in *Wac* mutants (Figure 7—figure supplement 4). *Fggy* is shown as an example of a metabolic gene with strong upregulation (Figure 7D). We also found a number of protocadherin genes (*Pcdhga6, Pcdhga8, Pcdhga9, and Pcdhga11*) were significantly upregulated (Figure 7B); *Pcdhga11* is shown which has consistent DE across sexes and samples (Figure 7D). We next tested for enrichment of disease associated genes using custom gene panels, finding no significant enrichment for high-confidence ASD genes based on rare mutations (Fu et al., 2022) (n=180) or the larger set of SFARI-associated ASD genes (Abrahams et al., 2013) (n=1137), nor was there enrichment for genes associated with microcephaly (n=1031), macrocephaly (n=347), and epilepsy (n=1318) (Piñero et al., 2020; Figure 7—figure supplement 4).

As DE analysis identified a potential sex bias with stronger effects in male *Wac* mutants (Figure 7A) and as the brain volume phenotype was larger in males (Figure 6A), we further investigated the sex-stratified DE results. Sex-stratified transcriptomic signatures were largely directionally correlated with each other, indicating most DEGs having a similar shift in RNA expression across sexes, with a large fraction of genes that had a stronger effect in males. (Figure 7—figure supplement 4). We used GSEA to identify functional terms that were activated (upregulated) or suppressed (downregulated) in mutants (Figure 7E). Terms identified in the GO analysis of the full dataset showed concordant enrichment in both sexes (RNA binding, RNA processing, perineuronal net, lipid import into cells), but with higher significance in males. Notably, the metabolic-related terms (purine-containing compound metabolic process, and carboxylic acid metabolic process) showed opposite directions with significant suppression in males. There was also concordant activation of dopaminergic neuron differentiation, integrin binding, developmental growth, chromatin organization, and regulation of cell cycle processes, with a stronger effect in males. Among the discordant signatures were suppression of neuron spine, chemokine binding, and regulation of cell migration in females and suppression of oxidative phosphorylation in males. Using a permutation test to assess altered ASD genes, male DEGs (*p*<0.05) showed a trend of enrichment for high-confidence ASD genes (*p*=0.07) where females did not (*p*=0.98) (data not shown). Examples of altered ASD transcripts are shown in Figure 7F and G, where males showed a unique alteration to several ASD genes compared to females. We highlight two ASD-associated genes with stronger effect in males versus females, *Prr12* (females, *p*=0.99; males, *p*=0.0006) and *Chd2* (females, *p*=0.39; males, *p*=0.0008) (Figure 7H). Finally, we wanted to validate if one of the more general changes between sexes in gene expression, that of the protocadherin alpha genes, was upregulated at the protein level. We thus probed P30 somatosensory cortices for pan protocadherin alpha via western blot (Figure 7I) as previously performed (Ing-Esteves et al., 2018); protein levels of protocadherin alpha were upregulated in the somatosensory cortices of *Wac* Hets (Figure 7J, *p*=0.003).

RNA processing and splicing were identified as perturbed biological processes in the enrichment analysis and the RNF20/40/WAC complex has been reported to play a role in regulating chromatin accessibility and transcription through H2B ubiquitination (Zhang and Yu, 2011), as well as mediating transcription elongation, and indirectly, splicing (Xu and Arnaout, 2002; Zhang and Yu, 2011). We performed event-based differential splicing (DS) analysis using the MAJIQ2.5 pipeline (Jinek et al., 2012). We found 13 DS junctions passing thresholds of 10% delta percent spliced in (dPSI) between WT and *Wac* Het, at 80% confidence level. As expected, we found two significant events representing floxed *Wac* exon skipping, and one intron retention in the Cre transgene locus Gt(ROSA)26Sor. We also detected skipped exons in *Snhg14*, alternative splice donors in *Dnmbp* and *Trmt61b*, alternative splicing acceptors in *Ssrp1* and *Trmt13*, retained introns in *Gm21992* and *Pigf*, and reduced intron retention in *Zc3h7a* (Figure 7—figure supplement 5, Supplementary file 6). None of these genes were identified as significantly differentially expressed. Overall, results of DE analysis capture modest transcriptional differences with evidence for stronger DE effects in males, as well as some evidence for differences in splicing events in the *Wac* Het mutants. Overall, our data shed new light on the transcriptomic mechanisms and phenotypes of *Wac* haploinsufficiency and provide further context for the existing studies of DESSH syndrome.

## Discussion

DESSH syndrome is a monogenic disorder that results in variable degrees of developmental delay/intellectual disability, craniofacial dysmorphic features, ophthalmological and gastrointestinal problems, hypotonia, and other neurological changes that may underlie the elevated co-diagnoses of ASD, ADHD, and seizures. Previous work identified distinct molecular and cellular changes, including alteration to the mTOR signaling pathway and nuclear regulation of transcription that may also overlap with our studies. A major limitation is that these previous studies were restricted to invertebrates and cell lines but not vertebrates. Thus, we sought to test if genetic depletion models of the *Wac* gene in vertebrates could recapitulate some of the reported symptoms in humans and further use our mouse model to probe novel anatomical and molecular changes for future studies. While the mouse and zebrafish models mostly recapitulated most core DESSH symptoms tested herein, they were not completely aligned. Importantly, mouse Hets recapitulated many DESSH symptoms while zebrafish *waca* KOs were needed to realize similar phenotypes. Since zebrafish have both *waca* and *wacb* genes, it is possible that some compensation occurs and only a complete deletion of one of the genes, here *waca*, could reveal symptoms that reflect DESSH patients.

While this study describes two novel vertebrate models for DESSH syndrome, the mechanisms underlying our findings still need to be elucidated. This will be done in future studies using conditional *Wac* deletion models that are predicted not to result in lethality due to limited deletion of *Wac* in distinct brain or other tissues. It is possible that some mechanisms might be conserved from previous studies examining *Wac* in cell lines and *drosophila* (David-Morrison et al., 2016; Zhang and Yu, 2011). We previously found that the WAC protein still localizes to the nucleus and has a speckled pattern in mammalian neurons (Rudolph et al., 2023), similar to the first study examining *Wac* in a cell line (Xu and Arnaout, 2002). Thus, a nuclear function for *Wac* is still highly likely and needs to be further tested. In addition, the WAC protein’s WW, coiled-coil, and disordered conserved domains are likely to mediate distinct adaptor complex properties and potential other regulatory functions that have yet to be uncovered.

Craniofacial dysmorphism and social behavior deficits are highly prevalent in DESSH, with craniofacial dysmorphism existing in almost 100% of those diagnosed with DESSH while autism behaviors are seen in roughly 30% of patients (Marwan Shinawi, personal communication), which were trending in mice and profound in zebrafish. We also found a robust sensitivity to a seizure-inducing drug in mice but not zebrafish. Together, each model revealed unique strengths and better connections to core DESSH symptoms. Despite these differences, each model showed impacts upon GABAergic neurons. These changes in GABAergic neuron markers may or may not underlie the common behavioral social changes in each model while the seizures may have a different underlying mechanism. Regardless, these data present two novel vertebrate DESSH models that could provide new insights into *Wac* loss-of-function and the impacts that this gene regulates upon brain function over an evolutionary timeline.

We and others have observed a decrease in PV expression but no loss of MGE-lineage interneurons in other syndromic mouse models like *Cntnap2* and other models where just PV protein levels alone were assessed (Paterno et al., 2021; Peñagarikano et al., 2011; Vogt et al., 2018), suggesting this may be a common feature in some monogenic syndromes with overlapping phenotypic features. Individuals with DESSH syndrome can have seizures (Alawadhi et al., 2021; DeSanto et al., 2015; Leonardi et al., 2020; Lugtenberg et al., 2016), and we found that subthreshold GABAA receptor inhibition elicited seizures in *Wac* Het mice. The reaction to PTZ suggests that their brains are shifted towards increased excitability. It is also possible that this more excitable brain may influence the expression of PV, as we and others have found that brain activity can alter PV expression (Malik et al., 2019; Patz et al., 2004). Thus, it is still unclear whether the loss of PV leads to seizures in our model or if the elevated brain excitability could lead to changes in PV expression. No matter the cause, others have noted the importance of PV expression on behaviors relevant to ASD (Wöhr et al., 2015), suggesting that approaches to restore PV expression may be a future therapeutic to alleviate some symptoms of DESSH syndrome. Moreover, syndromes like DESSH may additionally benefit in the future from stem cell-based therapies that supplement brains with inhibitory neurons, which is currently being tested to treat epilepsy and other conditions with an imbalance of inhibition (Bröer and Vogt, 2024; Yokomizo et al., 2026).

Some novel findings beyond phenotyping of our vertebrate models manifested that may be relevant for human symptoms. First, mutant male mice exhibit volumetric increases in several brain regions as adults, suggesting sex differences may arise in DESSH patients as they age and specific brain regions should be further explored. Zebrafish also have craniofacial extensions in the forebrain region. In addition, while only examined in mice thus far, transcripts changed in male Het mice encompassed more ASD risk genes, which may have future implications for understanding *Wac* as a regulator of other ASD risk genes in a sex-dependent manner.

Finally, our transcriptomic characterization of the *Wac* mouse model at P2 identified DE genes enriched in RNA processing and metabolism that may be future targets to understand underlying mechanisms. The magnitude of DE was stronger in males and although the signature was largely concordant in effect direction between the sexes, this could be indicative of a sex-based transcriptional response to reduced *Wac* dosage. Differential splicing signatures were comparatively more subtle, with 13 altered splicing events in 9 genes, including *snhg14*, an antisense transcript in the *Ube3a* locus, associated with Angelman and Prader-Willi syndrome. Of interest, considering various sex-specific phenotypes associated with *Wac* haploinsufficiency and DESSH, male *Wac* mutants had stronger transcriptional phenotypes and a significant enrichment for ASD-linked genes among DEGs, suggesting possible convergence of neurodevelopmental pathways. Our forebrain analyses raise questions regarding the cell-type-specific and developmental aspects of transcriptomic phenotypes, which could be addressed by single-cell methods and longitudinal studies in the future. Overall, our transcriptomic results support roles for *Wac* during neurodevelopment and gene expression. We generated new vertebrate models to assess DESSH syndrome and their role during development in divergent vertebrate species. These phenotypes are a first attempt at understanding this complex ultrarare disorder and offer initial tools to probe further biological mechanisms and therapeutics.

## Materials and methods

### Animals

#### Mouse model

We thank the Wellcome Trust Sanger Institute Mouse Genetics Project (Sanger MGP) and its funders for providing the conditional mutant mouse gamete (C57BL/6N-Wac<tm2 c(EUCOMM)Wtsi>/Wtsi). Funding information may be found at https://www.sanger.ac.uk/collaboration/mouse-resource-portal/ , associated primary phenotypic information at https://www.mousephenotype.org/ and data about the development of these technologies (Bradley et al., 2012; Pettitt et al., 2009; Ryder et al., 2013; Skarnes et al., 2011; White et al., 2013). Sperm harboring the *Wac* conditional allele was used to fertilize C57BL6/N donor eggs; progeny were then genotyped via polymerase chain reaction (PCR). We next bred *Beta actin-Cre* mice (Lewandoski et al., 1997) with *Wac^Flox^* mice to generate wild type (WT) and constitutive Het mice. After germline recombination, *Wac* Het mice were backcrossed and bred with CD-1 mice for at least three generations before being used for experiments. CD-1 outbred mice were used due to *Wac*’s importance for survival, as these mice are more robust and can generate and care for more progeny; we attempted to collect embryonic constitutive KOs as early as E12.5 but never collected a KO, suggesting early embryonic lethality. All murine experiments were performed under the approval of Michigan State University’s Campus Animal Resources.

#### Zebrafish model

Zebrafish (*Danio rerio*) were maintained at 28.5℃ with a 14 hr light/10 hr dark condition and fed three times a day. Zebrafish embryos were cultured in egg water (pH 7.4) and embryonic stages were determined by standard method (Kimmel et al., 1995). All zebrafish experiments were approved by the Institutional Animal Care and Use Committees (IACUC) of Chungnam National University (CNU-00866) and zebrafish were reared by standard lab protocols. For all assessments, males and females were tested. If no differences were noted, the data were combined for analyses. If differences were noted, then the data were separated and presented independently. For all experiments, both male and female animals were assessed but only reported as separate sexes if it became apparent that there were differences for specific phenotypes. Otherwise, all data were combined.

Zebrafish *waca* gene sequence was acquired from the NCBI database (*waca*: NM_199660.1). The primer for in vitro transcription of sgRNA was designed by 5′- TAATACGACTCACTATAGCTACTACAACTGCAGGACAGGTTTTAGAGCTAGAA –3′ for the *waca* target site. Primer contained 5′ T7 promoter and 15 nucleotides at 3′ complement to the universal primer. Template for in vitro transcription of the sgRNA to knockout *waca* was produced by PCR with primer 5′ – TAATACGACTCACTATAGGGAGGAAGGACTGGCACACGTTTTAGAGCTAGAA – 3′ (Jinek et al., 2012). In vitro transcription was carried out using PCR products and MaxiScript T7 Kit (Ambion). Cas9 expression vector pT3TS-nCas9n (Addgene), linearized with XbaI (NEB) and purified via DNA extraction kit (ELPIS). Cas9 mRNA was synthesized using mMESSAGE mMACHINE T3 Kit (Ambion) and tailed poly (A) with *E. coli* Poly (A) Polymerase (NEB). sgRNA of *waca* was injected with Cas9 mRNAs into 1 cell stage embryos.

### Antibodies

Antibodies and information are detailed in Table 2 below.

**Table 2.** General parameters of the bulk-seq brains analyzed. Columns indicate sample ID (ID), Genotype (WT = wild-type, HET = Wac mutant), Sex (M = Male, F = Female), RNA concentration, RNA integrity number (RIN) from bioanalyzer, A230/A260 value from spectrometer, number of aligned reads, and full sample metadata identifier (ID + Genotype + Sex).

Antibody (host/dilution)	Company	Category #	Clone	Lot #
Alpha-tubulin (mouse, 1:3000)	Abcam	ab7291	DM1-A	GR310199-6
Beta-catenin (rabbit, 1:4000)	Abcam	ab32572	E247	GR184212-83
CTIP2 (rat, 1:500)	Abcam	Ab18465	n/a	n/a
ERK1/2 (rabbit, 1:4000)	Cell Signaling	4695	137F5	35
pERK1/2^Thr202/Tyr204;Thr185/Tyr187^ (rabbit, 1:4000)	Cell Signaling	9101	n/a	31
GAD65/67 (rabbit, 1:4000)	Sigma-Aldrich	G5163	n/a	n/a
GAPDH (rabbit, 1:4000)	Cell Signaling	2118	14C10	14
Gephyrin (rabbit, 1:4000)	Cell Signaling	43928	D4J4R	1
IBA1 (goat, 1:500)	Cell Signaling	17198	E404W	1
LHX6 (mouse, 1:200)	Santa Cruz Bio.	sc-271433	A-9	B2219
NeuN (mouse, 1:400)	Millipore	MAB377	A60	3061189
OLIG2 (rabbit, 1:400)	Millipore	AB9610	n/a	3071572
Parvalbumin (rabbit, 1:400)	Swant	PV27	n/a	2014
Protocadherin alpha (rabbit, 1:3000)	SySy	190 003	n/a	1–8
PROX1 (rabbit, 1:500)	Abcam	ab199359	EPR19273	GR3216583
PSD95 (rabbit, 1:4000)	Cell Signaling	3450	D27E11	4
S100beta (rabbit, 1:500)	Proteintech	15146–1-AP	n/a	00092556
S6 (rabbit, 1:4000)	Cell Signaling	2217	5G10	7
pS6^Ser240/244^ (rabbit, 1:4000)	Cell Signaling	5364	D68F8	8
SATB2 (mouse, 1:500)	Abcam	Ab51502	n/a	n/a
Somatostatin (rat, 1:200)	Millipore	MAB354	YC7	2219310 A
TBR1 (rabbit, 1:500)	Abcam	Ab31940	n/a	n/a
mUb-H2B (rabbit, 1:4000)	Cell Signaling	5546	D11	6
WAC (rabbit, 1:4000)	Abcam	ab109486	n/a	GR175251-1

### Behavior (mouse)

Six to eight week aged mice of both sexes were tested; no differences were observed in any assay between sexes and data represent both groups. The persons performing the behaviors and analyses were blinded to the genotypes.

#### 3-chamber social interaction

During habituation, test mice had 10 min in the center of a rectangular chamber divided into 3-chambers by plexiglass containing small holes to navigate. Side chambers contained empty holders, with small holes allowing snout contact. Following initial habituation, an unfamiliar sex- and age-matched mouse (habituated to round holders/cages previously) was put in one of the chambers randomly, with doors of the lateral chambers closed and a novel object in the other. The test subject explored the apparatus for 10 min. Sniffing time was recorded using an overhead video camera and analyzed using ANY-Maze software. Time spent in each chamber and interaction with mouse/object was recorded.

#### Elevated plus maze

Individual mice were placed in the center of a two-open and two-enclosed-arm 50 cm height platform, facing an open arm (Kraeuter et al., 2019b). Mice were allowed to explore for 5 min while being tracked and later analyzed by ANY-maze software. The % open-arm entries [(total number of entries/number of open-arm entries)×100] and % time spent in the open arms were assessed.

#### Open field

Individual mice were placed in an open chamber with a center region designated by a 10×10 cm^2^ area and the remaining area to the wall designated as periphery (Kraeuter et al., 2019c). Mice roamed freely for 10 min while being tracked by an overhead camera. The total distance traveled and the mean speed were analyzed using ANY-maze software, as well as time spent in center versus periphery.

#### Radial arm water maze

Individual mice were placed in a corner of a circular swimming pool containing six swim arms radiating from a central area (Alamed et al., 2006); water temperature of 26 °C. A hidden escape platform located at the end of one arm; mice were allowed up to 60 s/trial (15 trials total) on day 1 to find the platform in a room with four distinct spatial cues mounted onto each of the four walls of the room. Each trial was followed by a 15 s rest period on the platform and each trial will alternate between having a visual cue on the platform and being hidden. If a mouse did not find the platform within 60 s, it was guided there and allowed the rest period. After learning trials, each mouse was dried with a small towel and allowed to rest for 30 min in a dry cage with a recirculating water blanket under half of the cage to provide warmth. On the second day, each mouse underwent 15 trials using only the spatial cues (hidden platform) and the number of errors (wrong arm entries) was recorded by an overhead camera and reported.

#### Y-maze

Individual mice were placed at the end of one arm of a Y-maze and allowed to explore for 5 min while being filmed by an overhead camera. Entries into all arms were noted (four paws need to be inside the arm for a valid entry) and a spontaneous alternation was counted if an animal enters three different arms consecutively. The percentage of spontaneous alternation were calculated according to the following formula: [(number of alternations) / (total number of arm entries − 2)]×100.

### Behavior (zebrafish)

#### Novel tank assay

Novel tank assay was performed as previously described (Choi et al., 2018; Kim et al., 2017; Park et al., 2019). 3 month/older WT and *waca* KO sibling zebrafish were tested in the behavior tank (24×15 ×15 cm). The back and sides were covered with non-transparent white paper. Behavior tests were recorded for 20 min using a video camera (Sony, HDRCX190). All fish were returned to the facility system tanks after completion of the behavioral tests. Recorded video files were analyzed using EthoVision XT7 software (Noldus).

#### Social cohesion test

Zebrafish group social cohesion was measured (Choi et al., 2018; Kim et al., 2017). During one session, 5 WT sibling or *waca* KO sibling zebrafish were placed in the behavior tank. Fish groups were recorded for 20 min using a video camera (Sony, HDRCX190). Videos were analyzed using 12 individual screenshots taken every 10 s for 3–5 min as early phase and 17–19 min as late phase. Distances between individual zebrafish in the group were measured using ImageJ for each screenshot and compiled.

### Cranio-facial analyses

#### Mouse model

The skulls were stained with Alizarin red for bone as previously published (Yen et al., 2010). Quantification of the suture and fontanel areas and the skull width was performed from photographs using ImageJ.

#### Zebrafish model

Zebrafish cartilage was stained with Alcian Blue (Sigma) as previously described (Dalcq et al., 2012; Pruvot et al., 2014). Zebrafish larvae at 13 dpf were fixed with 4% paraformaldehyde (PFA) overnight at 4 °C and treated with bleaching solution (3% H_2_O_2_/0.5% KOH in distilled water) for 30 min, rinsed out with Phosphate-buffered saline with 0.1% Tween 20 (PBST). Specimens were transferred to methanol to store at –20°C. Zebrafish cartilage was stained with Alcian blue solution (0.1% Alcian blue with 70% EtOH) for 1 hr and then dehydrated in 70% EtOH. Specimens were rinsed in 100% PBST and then transferred to 90% glycerol with 1% KOH to analyze on a dissecting microscope.

### Differential splicing (DS) analysis

Differential splicing (DS) analysis was performed using MAJIQ v2.5.1 (Vaquero-Garcia et al., 2023). In short, the *majiq build* module was executed on WT (n=14) and *Wac* Het (n=10) BAM files using GRCm38.95.gff3 genomic reference. DS quantification was performed using *majiq deltapsi* module, with two.majiq file groups delineating WT and Het samples. DS splicing was visualized using the *voila view* command with abs(E(dPSI)) threshold set to 0.1 and confidence threshold set to 0.8. Output files from the MAJIQ pipeline are deposited at Zenodo (https://doi.org/10.5281/zenodo.10999584).

### Electrophysiology

Coronal cortical slices (300 μm thick) were prepared (between postnatal ages 46 and 55 using methods previously described Crandall et al., 2017; Crandall et al., 2015). Briefly, mice were deeply anesthetized with isoflurane before decapitation. Brains were then quickly removed and placed in a cold (~4 °C) oxygenated slicing solution (95% O_2_, 5% CO_2_) containing (in mM): 3 KCl, 1.25 NaH_2_PO_4_, 10 MgSO_4_, 0.5 CaCl_2_, 26 NaHCO_3_, 10 glucose, and 234 sucrose. Slices were cut using a vibrating tissue microtome (Leica VT1200S) and then transferred into a chamber containing warm (32 °C) oxygenated (95% O_2_, 5% CO_2_) artificial cerebrospinal fluid (ACSF) containing (in mM): 126 NaCl, 3 KCl, 1.25 NaH_2_PO_4_, 2 MgSO_4_, 2 CaCl_2_, 26 NaHCO_3_, and 10 glucose. Slices were kept at 32 °C for 20 min followed by room temperature for an additional 40 min before recording.

For recordings, individual slices were transferred to a submersion recording chamber and continually perfused (~3 ml/min) with warm (32 °C) oxygenated (95% O_2_, 5% CO_2_) ACSF containing (in mM): 126 NaCl, 3 KCl, 1.25 NaH_2_PO_4_, 2 MgSO_4_, 2 CaCl_2_, 26 NaHCO_3_, and 10 glucose. Neurons were visualized using infrared differential interference contrast (IR-DIC) and fluorescence imaging using a Zeiss Axio Examiner.A1 microscope mounted with a video camera (Olympus XM10-IR) and a 40 x water-immersion objective. Whole-cell recordings were obtained from pyramidal neurons in layer 2/3 of the primary somatosensory cortex using borosilicate glass pipettes (4–6 MΩ tip resistance) containing a potassium-based internal solution (mM): 130 K-gluconate, 4 KCl, 2 NaCl, 10 HEPES, 0.2 EGTA, 4 ATP-Mg, 0.3 GTP-Tris, and 14 phosphocreatine-K (pH 7.25, 290 mOsm). All whole-cell recordings were corrected for a 14 mV liquid junction potential.

Data were recorded and digitized at 20 or 50 kHz using Molecular Devices hardware and software (MultiClamp 700B amplifier, Digidata 1550B4, and pClamp11.1). Signals were low-pass filtered at 10 kHz before digitizing. During the recordings, the pipette capacitances were neutralized, and series resistances (typically between 10 and 25 MΩ) were compensated online (100% for current-clamp, 70% for voltage-clamp). Series resistances were continually monitored throughout the recordings.

Analysis of electrophysiological data was performed in Molecular Devices and Microsoft Excel as described (Crandall et al., 2017). Resting membrane potentials (RMP, mV) were measured after break-in with no applied current. Input resistance (Rin, MΩ) was measured using Ohm’s law by measuring the voltage response from rest to an injection of a small negative current (15–25 pA). Membrane time constants (τm, ms) were measured from the average response (see Rin above) by fitting a single exponential to the initial falling phase of the response (100–300 ms; omitting the 1st ms). Membrane capacitance (Cin, pF) was calculated by τm/Rin. Rheobase currents (pA) were defined as the minimum positive current (5 pA steps) to elicit an AP from a holding potential of −84 mV. AP properties were measured from the first spike evoked by the rheobase current. AP threshold (mV) was defined as the membrane potential at which its first derivative (dV/dt) exceeded 10 mV/ms. AP amplitudes (mV) were defined as the voltage difference between the threshold and the peak of the AP. AP half-widths (ms) were measured at the half-height between the threshold and the AP peak. The max rate of rise and decay (mV/ms) was defined as the maximal dV/dt during the rising phase and falling phase of the same AP, respectively. Frequency-intensity (F/I) relationships were obtained by holding the soma at −84 mV with intracellular current and injecting suprathreshold positive current (50 pA steps, 500 ms duration). F/I slopes (Hz/pA) were determined using the initial frequency (reciprocal of the first interspike interval) over the entire F-I plot.

Spontaneous excitatory postsynaptic currents (sEPSCs) were recorded at –94 mV. Initially, individual events were manually selected and compiled to create an averaged sEPSC template that was then used to automatically detect similar events. Using this template, individual events were automatically selected by the software, but each event was also checked visually to confirm quality. Fifty sEPSCs were selected for each neuron for analysis of synaptic properties, including current amplitude and frequency. sEPSC amplitude and frequency were measured from events and automatically selected using our template. For each preselected event, the peak inward current amplitude was detected by the waveform template and was compared to a baseline immediately preceding each event.

### Genotyping

#### Mouse model

Primers to detect the recombined allele of the *Wac* genetic locus: Forward 5′-AGCTATGCGTGCTGTTGGG-3′ and Reverse 5′-CAAATCCCACAGTCCAATGC-3′. Thermocycling conditions were: 95 °C 3 min, (95 °C 30 s, 58 °C 30 s, and 72 °C 45 s for 35 cycles), 72 °C 3 min. Sanger sequencing to validate the recombined locus was performed by GeneWiz using the same primers on gel-purified PCR products.

#### Zebrafish model

Primers to detect the alleles of *waca*: Forward 5′- ATTTGAACCGGCAGATGATT-3′ and Reverse 5′-TCAGTGGGAGAAACCCAAAG-3′. Thermocycling conditions were: 95 °C 5 min, (95 °C 30 s, 60 °C 40 s, and 72 °C 20 s for 40 cycles), 72 °C 7 min and 15 °C 10 min. Primers to detect the genotypic alleles of *wacb*: Forward 5′-ATACCAGTCAAAGAGTCACTCAGCGAATGA-3′ and Reverse 5′-CACGTTCACCGGCCCCGTGAGAGAGACCAC-3′.

### Immunofluorescent staining

At P30, mice were transcardially perfused with phosphate-buffered saline (PBS), followed by 4% paraformaldehyde. The brains were then removed and postfixed in PFA for 30 min. Brains were transferred to 30% sucrose for cryoprotection overnight after fixation and then embedded in optimal cutting temperature compound before being coronally sectioned at 25 µm via cryostat. Sections were permeabilized in a wash of PBS with 0.3% Triton-X100, then blocked with the same solution containing 5% bovine serum albumin. Primary antibodies (details can be found in Table 2) were applied overnight at 4 °C, followed by three washes. Secondary antibodies (Alexa-conjugated 488 or 594 anti-rabbit or mouse, 1:300, Thermo Fisher) were applied for 1–2 hr at room temperature, followed by three washes and cover-slipped with Vectashield. Two to three images from the somatosensory cortex were acquired that contained all cortical layers. Cell counts were performed on these images using the cell counter function in Image-J software and images from the same animal were averaged to report a biological ‘n.’. Several hundred cells were counted per brain and 3–4 biological replicates were performed for each marker.

### MRI acquisition and analysis

High-resolution ex vivo MRI was conducted on brains that were transcardially perfused with 4% paraformaldehyde, using a 9.4T Bruker Advance (Bruker Biospin, Billerica, MA, USA) with the following parameters: TR/TE 3397/10.6, 10 evenly spaced echoes 10ms apart, matrix 1.75×1.25 (91 μm isotropic), and 30 slices 0.5 mm thick. T2-weighted imaging (T2WI) and T2 relaxation maps were computed as we previously described (Jullienne et al., 2020). T2WI scans were skull stripped (masking) using the segmentation tool from the ITK-SNAP software (version 3.8.0, RRID:SCR_002010) (Yushkevich et al., 2006). The T2WI mask was then used to generate a 3D reconstruction of the brain. T2WI parametric maps were generated and corrected for bias field inhomogeneities prior to registration. From T2WI data, brain and regional volumes and T2 relaxation times were obtained. The Australian Mouse Brain Mapping Consortium (AMBMC) atlas (Richards et al., 2011; Ullmann et al., 2013) was utilized to extract 40 bilateral regions using non-linear registration to each subject’s T2 images. Regional labels were applied using Advanced Normalization Tools (ANTs) and manually inspected for registration quality. Outliers were identified by calculating the interquartile range (IQR) for each regions, where value1.5IQR above the first quartile or below the third quartile were identified and excluded using Microsoft Excel.

### Microscopy

Mouse fluorescent images were acquired using a Leica DM2000 microscope with a mounted monochrome DFC3000G camera. Fluorescent images were adjusted for brightness/contrast and merged using Image-J software. The skulls were photographed with Nikon SMZ1500 stereomicroscope and Nikon DSRi1 camera. Zebrafish images were acquired using Leica TL 5000 microscope with DFC 7000T camera.

### PTZ-induced seizure assessments

One gram of Pentylenetetrazole (PTZ) powder was dissolved in 250 ml of PBS and injected intraperitoneally at 50 mg of PTZ per gram of mouse body weight. After injection, mice explored a clean cage for 20 min. Seizure severity was scored based on a modified Racine scale (Vogt et al., 2011): (1) Freezing or idle behavior; (2) Muscle twitches and/or rhythmic head nodding; (3) Tail arching over the mouse’s backside accompanied by the mouse assuming a hunched posture; (4) Forelimb clonus; (5) Tonic-clonic seizures with a recovery to normal behavior; (6) Uncontrolled running/jumping/hyperactivity; (7) Full body extension of limbs; (8) Death.

### RNA-sequencing and bioinformatics analysis

Twenty-four mouse brains (14 WT and 10 *Wac* HETs; 7, 7 and 5, 5, males, females, respectively) were collected at P2 following instant decapitation. Samples were snap-frozen on dry ice and stored at –80°C until RNA preparation. On the day of RNA isolation, left and right forebrain hemispheres were separated from the hindbrain and the total RNA was consistently isolated from the right forebrain hemisphere. Total RNA was obtained using Ambion RNAqueous Total RNA Isolation Kit (cat# AM1912) and assayed via Agilent RNA 6000 Nano Bioanalyzer kit/instrument. Sample RIN scores ranged from 6.9 to 9.8, with a mean RIN score of 9.4.

Poly-A-enriched mRNA libraries were prepared at Novogene using Illumina reagents. Libraries were sequenced using the Illumina NovaSeq 6000 S4 system, paired-end 150 (PE150) method. Reads were aligned to mouse genome (GRCm38/mm10) using STAR (version 2.5.4b) (Dobin et al., 2013), and gene counts were produced using featureCounts (Liao et al., 2014). On average, 50 million paired-end reads (PE) aligned per sample, with a range of 32–84 million reads.

Data quality was assessed using FastQC. FastQC: A Quality Control Tool for High Throughput Sequence Data. Available online at: http://www.bioinformatics.babraham.ac.uk/projects/fastqc/ and principal component analysis (PCA) was used to determine the presence of sample outliers. All 24 samples were qualified for the analysis. Raw RNA-seq fastq files and a gene count matrix is available on GEO (GSE264597).

Bioinformatic analysis was performed using R programming language version 4.2.1. R: A language and environment for statistical computing. R Foundation for Statistical Computing, Vienna, Austria. URL https://www.R-project.org/ and RStudio integrated development environment version 2023.06.0. RStudio: Integrated Development Environment for R. Posit Software, PBC, Boston, MA. URL http://www.posit.co/. Plots were generated using ggplot2 R package version 3.4.0 (Wickham, 2016). Heatmaps were generated using the pheatmap R package 1.0.12.

#### Differential expression (DE) analysis

For DE analysis, we used edgeR R package (Robinson et al., 2010). Genes with a minimum of one counts per million (CPM) in at least six samples were included in the analysis. The first surrogate variable from the SVA-batch-correction method was used as a covariate in the edgeR GLM model (Leek, 2011). For sex-stratified DE, we used a threshold of CPM >1 in at least three samples, and no batch correction. Reads Per Kilobase per Million mapped reads (RPKM) were used for plotting summary heatmaps and expression data of individual genes.

#### Gene ontology enrichment analysis

To test for enrichment of GO terms, we used the clusterProfiler R package version 4.14.6 (Bioconductor). Mouse Gene Ontology (GO) data for biological processes (BP), molecular function (MF), and cellular component (CC) were downloaded from Bioconductor (org.Mm.eg.db). For the analysis presented here, DE genes with a *p*-value <0.05 were used as input, and we required a minimum set of 5 and a maximum of 1000 as well, as at least two significant DE genes in a GO term. We applied a Benjamini-Hochberg (BH) correction on significance, a strategy recommended for gene set analysis that accounts for multiple testing comparisons. We reported terms with *p*-value <0.05. The set of DE genes was compared against the background set of genes expressed in our study based on minimum read-count cutoffs described above. To test for enrichment of GO terms, we used the fgsea R package version 1.32.4, via clusterProfiler. To control for transcriptional noise, we only used protein-coding transcripts with a logCPM >1. We tested against terms for BP, MF, and CC using a minimal gene set size of 5 and maximum of 1000. We again used a Benjamini-Hochberg (BH) correction on significance and reported terms with *p*<0.05. For the custom GSEA we pulled genes associated with epilepsy (C0016399), microcephaly (C1837501), and macrocephaly (C1836599) from DisGeNET (Piñero et al., 2020), SFARI genes from Abrahams et al., 2013, and high-confidence ASD genes from Fu et al., 2022 with a FDR <0.05.

#### Permutation test

To determine the significance of the overlap between DE genes identified in RNA-seq and disease gene sets, we used a permutation test. We used the DE genes with *p*<0.05 to compare against the ASD gene set from Fu et al., 2022 FDR <0.05 and the epilepsy gene set from DisGeNET (Piñero et al., 2020). To generate a null distribution, we performed 10,000 permutations. In each iteration, a random set of genes equal in size to the DE gene list was sampled without replacement from the background gene set of those tested in DE analysis. The overlap between the disease gene set and each permuted gene set was recorded. A p-value was calculated as the proportion of permutations in which the overlap was greater than or equal to the observed overlap.

Analysis code is available from GitHub (copy archived at NordNeurogenomicsLab, 2026).

### Western blots

P30 somatosensory cortices were dissected and frozen on dry ice. Next, they were lysed in RIPA buffer containing protease and phosphatase inhibitors and combined with Laemmli buffer containing 2-Mercaptoethanol and incubated at 95 °C for 5 min to denature the proteins. Equal amounts of protein lysates were separated on 10% SDS-PAGE gels and then transferred to nitrocellulose membranes. The membranes were washed in Tris-buffered saline with Tween-20 (TBST) and then blocked for 1 hr in TBST containing 5% non-fat dry milk (blotto, sc-2324 Santa Cruz Biotechnology). Membranes were then incubated with primary antibodies overnight at 4 °C, washed three times with TBST, incubated with secondary antibodies for 1 hr at room temperature and then washed three more times with TBST. Membranes were next incubated in ECL solution (BioRad Clarity substrate 1705061) for 5 min and chemiluminescent images obtained using a BioRad Chemidoc MP imaging system. Antibody details can be found in Table 2, and uncropped western blots are provided as source files associated with each figure.

### Whole-mount in situ hybridization (WISH)

WISH was performed using DIG-labeled antisense RNA probes for *gad1b*. RNA probes were synthesized using DIG-RNA labeling kit (Roche) (Thisse and Thisse, 2008). Zebrafish larvae were fixed in 4% PFA in PBS overnight at 4 °C then dehydrated with 100% Methanol. Larvae were rehydrated with 0.1% Diethyl pyrocarbonate with PBST (DEPC-PBST, Sigma Aldrich). Larvae were permeabilized with 10 μg/ml proteinase K (Roche) according to developmental stage. Larvae were re-fixed for 15 min in 4% PFA, washed with DEPC-treated PBST, and pre-hybridized in HYB solution (50% formamide, 5 X saline sodium citrate, 50 μg/ml heparin, 500 µg/ml torula RNA, 46 mM citric acid pH 6.0, 0.1% Tween20) for 1 hr at 70 °C. Antisense DIG-labeled RNA probes were added in HYB solution and incubated overnight at 70 °C. Larvae were washed in a preheated mixture of 50% saline sodium citrate containing 0.1% Tween-20 and 50% hybridization solution at 70 °C. Larvae were blocked and then incubated with 1/4000 concentration of anti-DIG Fab fragment conjugated with alkaline phosphatase (Roche) overnight at 4 °C. Larvae were washed in PBST, then incubated in staining solution, including NBT/BCIP as alkaline phosphatase substrate in the dark until sufficient staining appeared. Larvae were mounted in 90% glycerol in PBST and were visualized via dissecting microscope.

### Quantification and statistical analysis

Statistical analyses were performed using Prism versions 7 and 10, a *p*-value of <0.05 was considered significant. For parametric measures of two groups, a two-tailed t-test was performed; parametric measures of three or more groups utilized a ONE-Way ANOVA with Tukey post-test. For the male retropleinal cortex, male parietal cortex, and female corpus callosum MRI data, the data were not distributed normally, and we used Mann–Whitney test. If other statistical analyses were used, they are denoted in the results or figure legends.

## Data Availability

RNA-sequencing data were performed and are embedded in the manuscript files.

## Additional files

Supplementary file 1. Surrogate variable analysis (SVA)-corrected differential expression analysis of *Wac* mutants; combined sexes. Genes are annotated by common name and ENSEMBL gene ID. Effect size indicated by log fold change (logFC) and relative expression by log counts per million (logCPM). Significance is reported by a likelihood ratio test statistic (LR), standard *p*-value (*p*-value), and the multiple-testing corrected *p*-value or false discovery rate (FDR).

Supplementary file 2. Differential expression analysis of *Wac* mutants, using only male samples. Genes are annotated by both common name and ENSEMBL gene ID. Effect size is indicated by log fold change (logFC) and relative expression by log counts per million (logCPM). Significance is reported by a likelihood ratio test statistic (LR), standard p-value (*p*-value), and the multiple-testing corrected p-value or false discovery rate (FDR).

Supplementary file 3. Differential expression analysis of *Wac* mutants, using only female samples. Genes are annotated by both common name and ENSEMBL gene ID. Effect size is indicated by log fold change (logFC) and relative expression by log counts per million (logCPM). Significance is reported by a likelihood ratio test statistic (LR), standard *p*-value (PValue), and the multiple-testing corrected *p*-value or false discovery rate (FDR).

Supplementary file 4. Gene Ontology (GO) over representation analysis of differential expression results for different models (Full, Male, Female). Ontology indicates the source of the term set (MF = Molecular Function, BP = Biological Processes, CC = Cellular Component). GO terms are identified by GO:ID number and term description. GeneRatio is the ratio of differentially expressed genes associated with the term to the total number of differentially expressed genes tested. BgRatio is the ratio of background genes associated with the term to the total number of genes in the background set. RichFactor is the ratio of observed differentially expressed genes to expected genes for the term (GeneRatio ÷ BgRatio). Fold enrichment of differentially expressed genes for the term relative to background expectation. The zscore, or standardized enrichment score reflecting deviation from expected overlap. Significance is indicated by the p-value from a Fisher’s exact test, multiple-testing–corrected *p*-value (p.adjust) using a Benjamini–Hochberg false discovery rate correction, and false discovery rate (q-value). geneID shows the differentially expressed genes in the GO term. DE indicates direction of differential expression in the model. Model indicates the differential expression model (Full = SVA-corrected full dataset, Male = male-only differential expression, Female = female-only differential expression).

Supplementary file 5. Gene ontology (GO) enrichment analysis using fgsea of differential expression results for different models (Full, Male, Female). Ontology indicates the source of the term set (MF = Molecular Function, BP = Biological Processes, CC = Cellular Component). GO terms are identified by GO:ID number and term description. SetSize indicates the number of genes from the gene set that are present in the ranked gene list used for GSEA. EnrichmentScore is the raw gene set enrichment score reflecting the degree to which genes in the set are overrepresented at the top or bottom of the ranked list. The normalized enrichment score (NES), which accounts for gene set size and allows comparison across gene sets. Significance is indicated by the p-value from a Fisher’s exact test, multiple-testing–corrected *p*-value (p.adjust) using a Benjamini–Hochberg false discovery rate correction, and false discovery rate (q value). Rank position at which the maximum enrichment score is observed in the ranked gene list. The leading edge is the subset of genes from the gene set that contribute most strongly to the enrichment signal. Core enrichment, an indicator or list specifying genes belonging to the leading-edge subset. Model indicates the differential expression model (Full = SVA-corrected full dataset, Male = male-only differential expression, Female = female-only differential expression).

Supplementary file 6. MAJIQ-based alternative splicing analysis of *Wac* mutants. Local splicing variations (LSVs) are reported per gene and junction. Genes are annotated by Ensembl gene ID. Splicing effect size is quantified as the mean change in percent spliced in (mean ΔPSI) per LSV junction. Statistical confidence is reported as the posterior probability that the splicing event is changing (probability_changing) or non-changing (probability_non_changing). Mean PSI values are provided separately for wild-type (WT_mean_psi) and heterozygous mutants (Het_mean_psi). Additional structural annotations include the number of junctions and exons per LSV, genomic coordinates of junctions, and intron retention coordinates where applicable.

MDAR checklist
