# Supplementary material for: Complementary vertebrate Wac models exhibit phenotypes relevant to DeSanto-Shinawi Syndrome
Source: eLife. 2026 Sep 3;14:RP109104. doi: 10.7554/eLife.109104 (PMC13541301; doi:10.7554/eLife.109104)
Supplement: Supplementary file 1 [file elife-109104-fig5-data1.pdf]

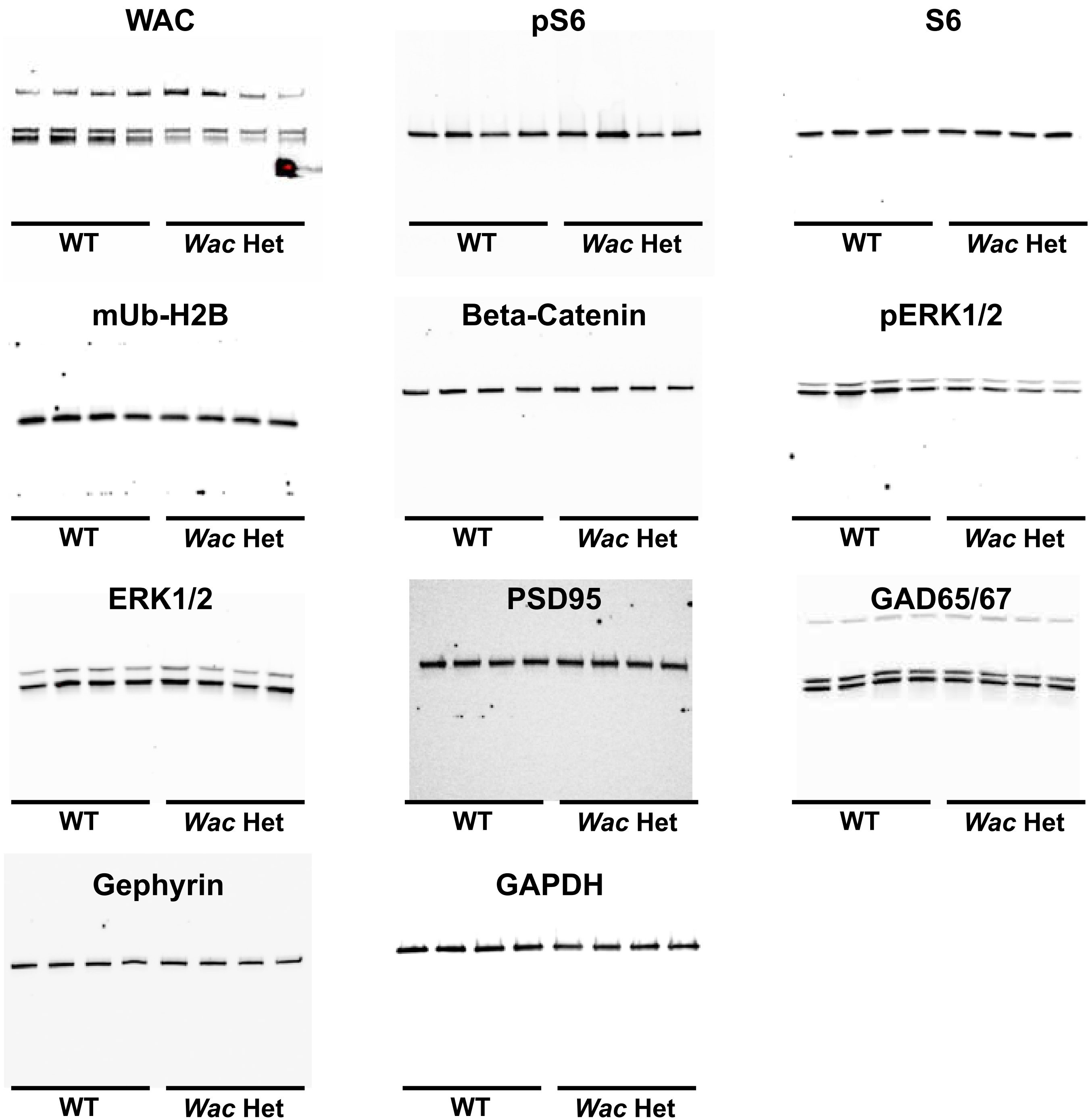
WAC
pS6
S6
WT
Wac Het
mUb-H2B
Beta-Catenin
pERK1/2
ERK1/2
PSD95
GAD65/67
Gephyrin
GAPDH
