# Supplementary material for: Complementary vertebrate Wac models exhibit phenotypes relevant to DeSanto-Shinawi Syndrome
Source: eLife. 2026 Sep 3;14:RP109104. doi: 10.7554/eLife.109104 (PMC13541301; doi:10.7554/eLife.109104)
Supplement: Supplementary file 2 [file elife-109104-fig7-data1.pdf]

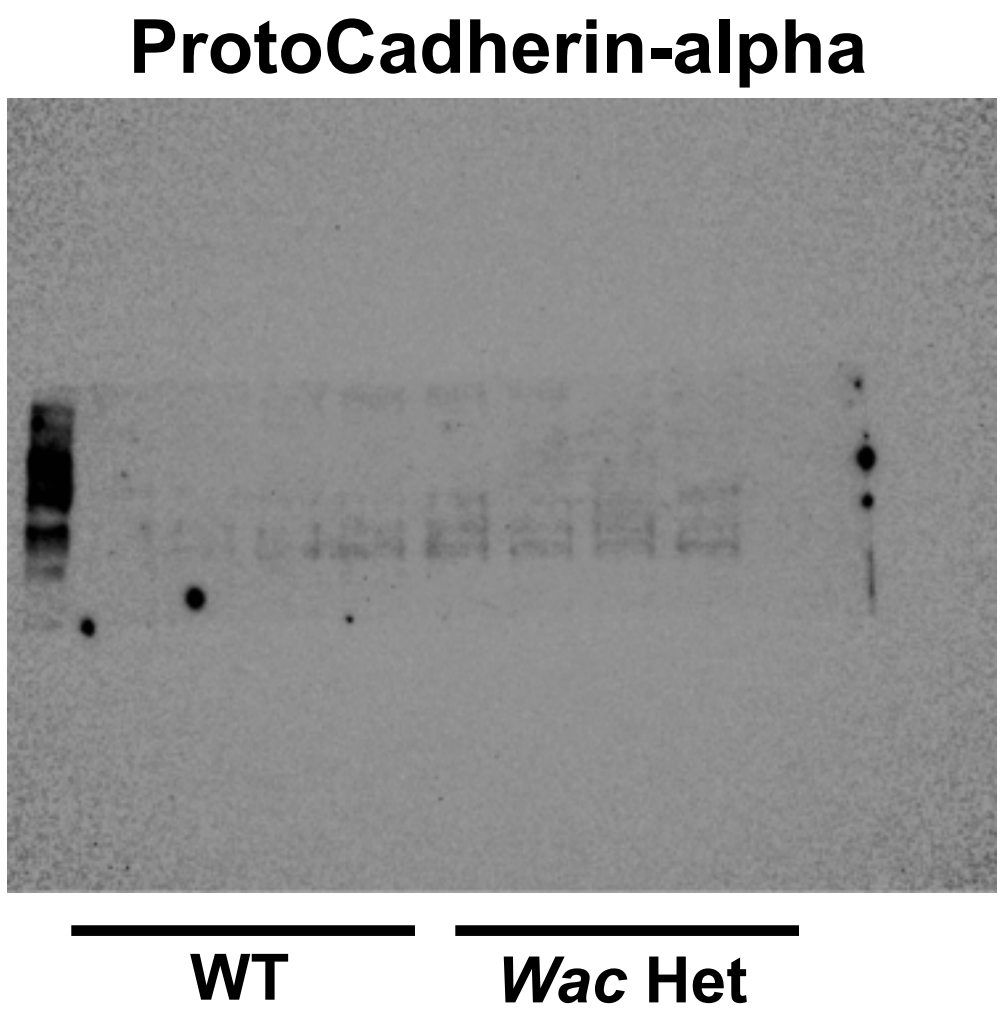
ProtoCadherin-alpha
WT
Wac Het

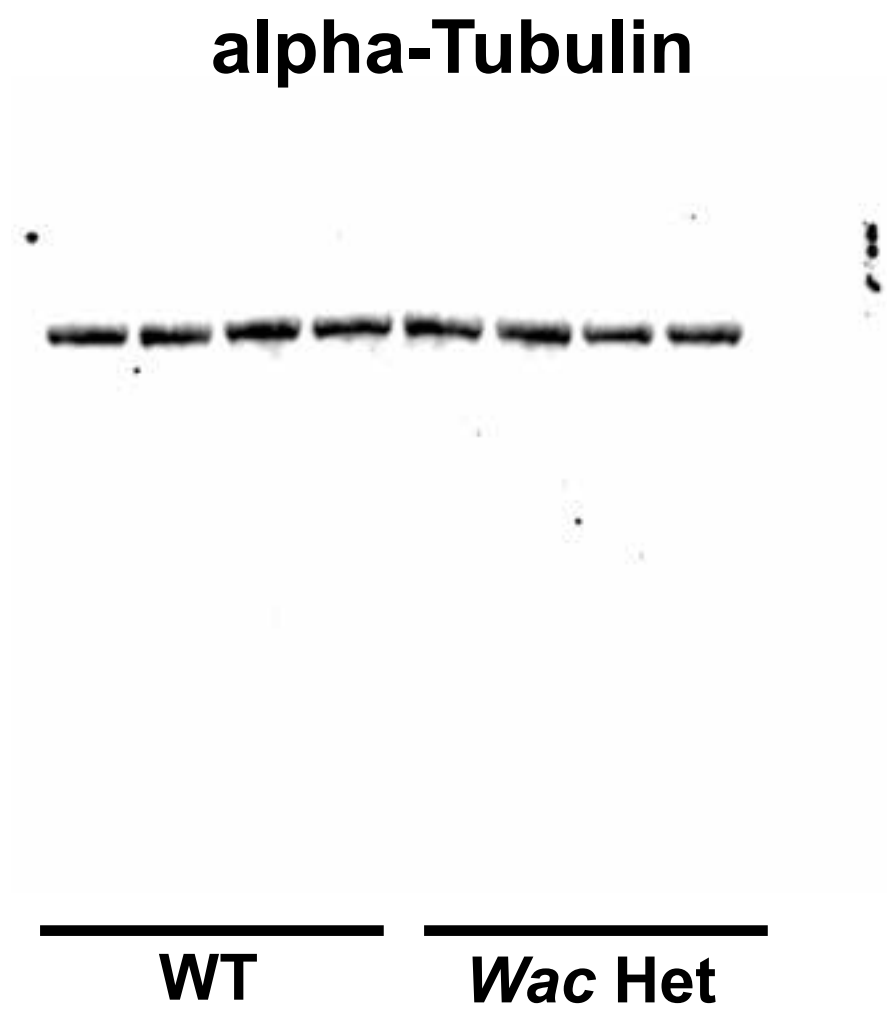
alpha-Tubulin
WT
Wac Het
