# Supplementary material for: Complementary vertebrate Wac models exhibit phenotypes relevant to DeSanto-Shinawi Syndrome
Source: eLife. 2026 Sep 3;14:RP109104. doi: 10.7554/eLife.109104 (PMC13541301; doi:10.7554/eLife.109104)
Supplement: Peer review history

# Editor report: eLife Assessment

**DOI:** 10.7554/eLife.109104.3.sa0

**Authors:** Anne E West

**Affiliations:** Duke University United States

**Keywords:** Convincing, Important

This **important** study establishes the first vertebrate models of DeSanto-Shinawi Syndrome, revealing conserved craniofacial and social and behavioral phenotypes across mouse and zebrafish that mirror key clinical features. The **convincing** evidence is supported by behavioral, anatomical, and molecular analyses of Wac animal mutants. This study sets a baseline for future mechanistic studies and reports a platform to test approaches to reverse phenotypes.

# Referee report: Reviewer #1 (Public review)

**DOI:** 10.7554/eLife.109104.3.sa1

[Editors' note: this version has been assessed by the Reviewing Editor without further input from the original reviewers. The authors have addressed the comments raised in the previous round of review.]

Summary:

The authors generated mouse and zebrafish models for DeSanto-Shinawi Syndrome, caused by loss-of-function variants in the WAC gene. Using these vertebrate systems, they demonstrate conserved craniofacial and social-behavioral phenotypes that parallel human clinical features, along with deficits in GABAergic markers. They observe increased seizure susceptibility and male-biased brain volumetric changes in Wac mutant mice. Together, these findings begin to define the biological consequences of Wac haploinsufficiency and provide valuable resources for future mechanistic studies.

Strengths:

WAC is a high-confidence neurodevelopmental disorder gene and one of the genes identified by large-scale exome sequencing efforts, including the Satterstrom et al. (2020) autism spectrum disorder cohort. This study establishes the first vertebrate Wac models, addressing a major gap in the understanding of DeSanto-Shinawi Syndrome, and provides a framework for studying other syndromic forms of autism. The models generated will be impactful and useful to the community to study and understand DeSanto-Shinawi Syndrome.

The cross-species analysis is important and well executed, and reveals both conserved and divergent phenotypes. The behavioral and anatomical assays are rigorously executed and well-controlled, and the inclusion of RNA-sequencing analyses adds valuable insights into the mechanisms underlying brain function in Wac mutants. Notably, the RNA-seq data reveal upregulation of several clustered protocadherins, genes central to neuronal identity and cell-cell interactions, which are known to be regulated by dynamic developmental regulation of chromatin architecture. This observation provides an intriguing hint that could link Wac function to higher-order chromatin organization and neuronal connectivity.

Weaknesses:

The evidence is solid, though the study remains incomplete in its mechanistic depth and molecular interpretation. The authors compellingly describe behavioral, anatomical, and transcriptomic phenotypes associated with WAC loss, yet do not explore how WAC mechanistically regulates chromatin or transcription. Given prior evidence that WAC interacts with the RNF20/40 ubiquitin ligase complex and promotes histone H2B ubiquitination and transcriptional elongation, the paper would benefit from a discussion of these functions as a potential link between Wac haploinsufficiency and the observed changes in neuronal gene expression. Similarly, the authors mention WAC's WW and coiled-coil domains but do not consider how these domains could mediate nuclear interactions or recruitment of transcriptional cofactors that shape gene regulation and chromatin organization in neurons.

The transcriptomic analysis is rich but largely descriptive. Although the upregulation of clustered protocadherins is particularly intriguing, these findings are not validated or localized to specific neuronal populations. The study would be strengthened by independently validating the most significant RNA-seq changes, such as protocadherin gamma genes, using in situ hybridization methods to confirm the spatial and cellular specificity of expression changes.

# Referee report: Reviewer #2 (Public review)

**DOI:** 10.7554/eLife.109104.3.sa2

The authors describe the first deep neurological characterization of WAC mutation in two vertebrate species (zebrafish and mouse). They examine these at various levels, guided by the work in humans that has associated a heterozygous WAC mutation with DeSantos Shinawi Syndrome (DESSH). Therefore, they investigate the animals for a variety of phenotypes, following a template for what is seen when characterizing a new mouse/fish model of a developmental disability gene. Investigations include analysis of skull and jaw for abnormalities(both species), MRI of brain structure(in mice), electrophysiology(mice), assessment of signaling pathways (by Western blot, in mice), cell counts (both, more in mice), transcriptomics (mice), and behavior (both).

Generally, this describes an important first characterization of the consequences of the mutation. Most of the studies appear well-conducted and reasonably powered, thus solid or convincing.

# Author comment: Author response

**DOI:** 10.7554/eLife.109104.3.sa3

**Authors:** Kang-Han Lee, April M Stafford, Maria Pacheco-Vergara, Karol Cichewicz, Cesar P Canales, Nicolas Seban, Melissa Corea, Darlene Rahbarian, Kelly E Bonekamp, Grant R Gillie, Dariangelly Pacheco-Cruz, Alyssa M Gill, Hye-Eun Hwang, Yeong-Eun Kim, Katie L Uhl, Tara E Jager, Marwan Shinawi, Xiaopeng Li, Andre Obenaus, Shane R Crandall, Juhee Jeong, Alex S Nord, Cheol-Hee Kim, Daniel Vogt

**Affiliations:** Department of Biology, Chungnam National University Daejeon Republic of Korea; Michigan State University Grand Rapids United States; Department of Molecular Pathology, New York University College of Dentistry New York United States; University of California, Davis Davis United States; University of California, Davis Davis United States; Department of Psychiatry and Behavioral Sciences, University of California Davis Davis United States; Department of Psychiatry and Behavioral Sciences, University of California Davis Davis United States; Department of Psychiatry and Behavioral Sciences, University of California Davis Davis United States; Department of Physiology, Michigan State University East Lansing United States; Department of Physiology, Michigan State University East Lansing United States; Depertment of Pediatrics and Human Development, Michigan State University Grand Rapids United States; College of Human Medicine, Michigan State University Grand Rapids United States; Department of Biology, Chungnam National University Daejeon Republic of Korea; Chungnam National University Daejeon Republic of Korea; Depertment of Pediatrics and Human Development, Michigan State University Grand Rapids United States; Corewell Health Grand Rapids United States; Division of Genetics and Genomic Medicine, Department of Pediatrics, Washington University School of Medicine St. Louis United States; Depertment of Pediatrics and Human Development, Michigan State University Grand Rapids United States; Preclinical and Translational Imaging Center, University of California Irvine Irvine United States; Michigan State University Grand Rapids United States; New York University New York United States; University of California, Davis Davis United States; Chungnam National University Daejeon Korea (South), Republic of; Michigan State University Grand Rapids United States

The following is the authors’ response to the original reviews.

**Public Reviews:**

**Reviewer #1 (Public review):**

Summary:

The authors generated mouse and zebrafish models for DeSanto-Shinawi Syndrome, caused by loss-of-function variants in the WAC gene. Using these vertebrate systems, they demonstrate conserved craniofacial and social-behavioral phenotypes that parallel human clinical features, along with deficits in GABAergic markers. They observe increased seizure susceptibility and male-biased brain volumetric changes in Wac mutant mice. Together, these findings begin to define the biological consequences of Wac haploinsufficiency and provide valuable resources for future mechanistic studies.

Strengths:

WAC is a high-confidence neurodevelopmental disorder gene and one of the genes identified by large-scale exome sequencing efforts, including the Satterstrom et al. (2020) autism spectrum disorder cohort. This study establishes the first vertebrate Wac models, addressing a major gap in the understanding of DeSanto-Shinawi Syndrome, and provides a framework for studying other syndromic forms of autism. The models generated will be impactful and useful to the community to study and understand DeSanto-Shinawi Syndrome.

The cross-species analysis is important and well executed, and reveals both conserved and divergent phenotypes. The behavioral and anatomical assays are rigorously executed and well-controlled, and the inclusion of RNA-sequencing analyses adds valuable insights into the mechanisms underlying brain function in Wac mutants. Notably, the RNA-seq data reveal upregulation of several clustered protocadherins, genes central to neuronal identity and cell-cell interactions, which are known to be regulated by dynamic developmental regulation of chromatin architecture. This observation provides an intriguing hint that could link Wac function to higher-order chromatin organization and neuronal connectivity.

Weaknesses:

The evidence is solid, but the study remains incomplete in its mechanistic depth and molecular interpretation. The authors compellingly describe behavioral, anatomical, and transcriptomic phenotypes associated with WAC loss, yet do not explore how WAC mechanistically regulates chromatin or transcription. Given prior evidence that WAC interacts with the RNF20/40 ubiquitin ligase complex and promotes histone H2B ubiquitination and transcriptional elongation, the paper would benefit from a discussion of these functions as a potential link between Wac haploinsufficiency and the observed changes in neuronal gene expression. Similarly, the authors mention WAC's WW and coiled-coil domains but do not consider how these domains could mediate nuclear interactions or recruitment of transcriptional cofactors that shape gene regulation and chromatin organization in neurons.

We agree that many mechanisms underlying how both animal model phenotypes and human symptoms that are caused by the Wac gene still need to be worked out. Due to the need to generate a great deal of data to first describe these models in this manuscript this will be expanded upon later. In lieu of this, we plan to follow up with mechanistic papers later to fully address the gap that remains. We have now added a paragraph in the discussion to bring up these important points regarding the roles of Wac during transcription and how its protein domains might be involved in these processes.

The transcriptomic analysis is rich but largely descriptive. Although the upregulation of clustered protocadherins is particularly intriguing, these findings are not validated or localized to specific neuronal populations. The study would be strengthened by independently validating the most significant RNA-seq changes, such as protocadherin gamma genes, using in situ hybridization methods to confirm the spatial and cellular specificity of expression changes.

We have greatly expanded the analyses of the bulk RNA-seq data, including a more rigorous look into the differences in gene expression between sexes, which has additionally revealed males to be more impacted by Wac loss of function. We have also added new western blot data for pan protocadherin alpha, which is now validated to be upregulated in the cortex (new Figure 7I and 7J). We are holding back any additional data from this report as we have single nucleus RNA-seq data that will be reported on in follow-up papers with targeted conditional deletion models.

Finally, while the behavioral and MRI results add valuable breadth, their interpretation would be improved by clearer reporting of sample sizes, statistical corrections, and effect sizes to support claims of sex-specific and regional brain volume differences.

Some additional details have been added to the methods section. In addition, we have now provided sample sizes assessed in each figure legend.

**Reviewer #2 (Public review):**

The authors describe the first deep neurological characterization of WAC mutation in two vertebrate species (zebrafish and mouse). They examine these at various levels, guided by the work in humans that has associated a heterozygous WAC mutation with DeSantos Shinawi Syndrome (DESSH). Therefore, they investigate the animals for a variety of phenotypes, following a template for what is seen when characterizing a new mouse/fish model of a developmental disability gene. Investigations include analysis of skull and jaw for abnormalities(both species), MRI of brain structure(in mice), electrophysiology(mice), assessment of signaling pathways (by Western blot, in mice), cell counts (both, more in mice), transcriptomics (mice), and behavior (both).

Generally, this describes an important first characterization of the consequences of the mutation. Most of the studies appear well-conducted and reasonably powered, thus solid or convincing. However, there are a few places where the data presentation could be improved for clarity, and a few concerns about some choices in analytical approach for a couple of the experiments, where improved statistical approaches could improve their sensitivity and/or better rule out false positives, and thus the support of some of these claims is currently incomplete. There is also some lack of clarity about the rationale for some decisions regarding the fish genetics. Nonetheless, this is an important and useful first characterization of many phenotypes of these lines. Such experiments form a baseline for future mechanistic studies in the same lines and a platform to test approaches to reverse phenotypes.

Individual claims and their strength & weaknesses:

(1) The authors developed mouse and zebrafish models of WAC deletion

They used the existing KOMP floxed WAC line to generate a null allele. For the mouse, there is a Western showing that it is indeed null for the protein. The fish data is less robustly validated - they don't confirm the allele in null at the protein or RNA level, and fish have two paralogs (waca and wacb), and this paper only characterizes one of these. So this evidence is less clear. The evaluated mice are heterozygous (Het), similar to patients, while the fish appear to be evaluated as homozygous mutants.

We agree with the reviewer’s comments on zebrafish genetics. Since antibodies against zebrafish Wac proteins are not available, we could not examine protein levels in zebrafish. We predicted frameshift mutations due to DNA analyses in waca and wacb KO zebrafish. We made waca KO, wacb KO, and waca/wacb double KO zebrafish. waca/wacb double KO zebrafish showed a lethal phenotype, similar to homozygous mice mutants. Since wacb KO zebrafish did not show any detectable phenotype we do not report those here. However, we now show examples of the wacb and dKO zebrafish in Figure S1. Since waca KO zebrafish showed craniofacial and behavioral phenotypes that are comparable to mice Het and human patients, they are focused on in this report.

(2) The authors show that both species show altered craniofacial features

These data appear well powered, and the findings are robust.

We appreciate this confirmation.

(3) Each model altered GABAergic neurons

In mice, the authors stained with PV antibodies and saw a decrease in cells positive for this staining. A second marker, Lhx6, does not show a difference, suggesting this might be a change in PV expression rather than cell number. They could maybe look into the literature to see if this loss of just the protein also occurs in other models. Overall, the sample size here is a bit smaller than other parts of the paper (n=3), and the methods on the cell counts were less clear, so it is not as clear that this finding is as robust. The authors counted several other broad classes of cells, and those appear normal. Interestingly, there might also be some TBR1 mislocalization in layer 6 that might be significant with added power.

Thank you for these suggestions. Yes, other models also show this lack of PV expression even when MGE-lineage interneurons are present at normal levels. We mention in the discussion a previous study on the ASD gene CTNNAP2 that showed this. We also agree that there is a trend going on in the Tbr1 population. We assessed another WT and Het pair for Tbr1 laminar distribution and were able to determine that these changes held up and are now significantly different; the person counting these numbers was blind to the genotypes. Finally, we added more details to the methods to describe how the counting was performed.

The fish data is based on an in situ hybridization for GAD. The measure shown is the width of the positive area in the forebrain. This measure is not one I have seen much before, and has potential to be driven by something unrelated to GABA (e.g., if the whole forebrain were simply a bit smaller). So this analysis could use a couple of other approaches (density of signal?) and/or a control probe for some other brain gene showing the measure is normal, and thus it is not just a size issue.

To compare altered GABAergic neurons in mice and zebrafish, we tried to isolate zebrafish PV genes and examined their expression by whole-mount in situ hybridization, now included Figure S3 but found no differences. However, we could not find any zebrafish PV gene useful for GABAergic neurons. We chose to examine gad1b expression in the positive area of the forebrain in WT and waca KO zebrafish and then found differences in the brain area with gad1b expression. Since WT and waca KO brain sizes are generally the same we believe this measurement is reasonable to make this conclusion and have added text to the results section to justify.

(4) Mice were more susceptible to the seizure-inducing agent PTZ

These data appear well powered, and the findings are robust. The authors also did a fair amount of useful electrophysiology that was all normal, but appeared to be well executed.

Thank you, we appreciate this confirmation.

(5) Mice had changes in brain volume that interact with sex

The authors conducted an MRI on a good number of mice and reported a slight increase in global volume just in males. Sample size is fair, but the statistical approach here may be better if it puts males and females in the same model (to boost power and explicitly test for sex by genotype interaction that they report), and there is some chance that the brain region level differences that they report could include some false positives. They tested many regions, and it is not clear whether or not they corrected for the number of tests. Often, an FDR correction would be used in such imaging studies. It may be that only the most robust regional findings will survive those corrections. It is interesting data either way, but the analysis could be improved.

Given the 80 regions (bilaterally) that we used and the number of mice, i.e. 6-7, we are underpowered to robustly undertake FDR types of corrections. In the data presented we used t-tests between sex and regions to illuminate putative regional changes. However, we did revisit our MRI data and found three data sets where the results were not normally distributed. We thus changed our statistical test to Mann Whitney for male retrosplenial cortex, male parietal cortex and female corpus callosum, which are now reflected in the figures and differential statistics noted in figure legends.

(6) Several behaviors are altered in the mice as well

These studies were fairly well-powered (n=15,16), and they found several positive and negative results, including alterations in memory and sociability in both species. There is a minor statistical flaw in the three-chamber analysis they don't actually compare the Hets directly to the wildtypes in their statistical testing - a common mistake in neuroscience that should be addressed. But the data look like they will probably still be significant when correctly analyzed. In the supplement, the authors could do a bit more with the data they have to look at hyperactivity (i.e., show total motion in open field, not just time in center vs. periphery), and adding sex to their model might improve sensitivity for genotype effects.

Thank you for these suggestions. We have done several things to address this behavioral paradigm. First, we added more n’s and also switched from comparing the mouse vs. object to just comparing genotypes as a variable. In addition, we switched to quantifying a discrimination index, described in Phiilips et al., 2019 PMID: 31112129 for our measurement. These new data are shown in Figure 3A. Open field total distance traveled has now been added to Figure S2A. For all other measurements, we did first assess for sex differences but found none and thus compiled both sexes for the graphs.

(7) Some biochemical signaling pathways are altered in the brain

These are n=4 immunoblots, and show altered phospho ERK, but no changes in other signaling events predicted from prior WAC literature like H2B ubiquitination. They appear well done, and the authors share the full blots in the supplement.

Thank you, we appreciate this confirmation. Since Wac is an adaptor protein we needed to test these reported molecular changes in neurons that were previously only reported in cell lines and drosophila. We were not surprised that some of these previously reported changes would not be the same in brain cells. However, it is possible that these changes might arise in more discrete brain regions or at different times during development, which will be tested in our future conditional knockout models.

(8) WAC deletion also alters gene expression in the brain

These studies were well-powered for RNAseq, with 10 and 14 samples, using neonates (P2), just the forebrain. The sequencing quality metrics all looked good, and the approach to analysis was okay. It would be stronger to again include sex in the model, rather than separate by sex. There were some typos in this part of the paper that made part of the conclusions unclear, but the RNAseq nicely confirmed the mutation of the mice, and discovered many differentially expressed genes, consistent with the role of this gene as a regulator of transcription. The presentation could be expanded to make more use of the data. Overall, though, this is a useful first characterization of the transcriptome in the line.

Thank you for the suggestions. We have greatly expanded our assessments of the RNA-seq data. Upon analyzation of the data we found many differences between males and females and now show combined and sex-separated data. Our new data isolate several more extreme and some unique changes in males that are better shown as stand alone figure panels. In addition to these edits, we have also reworked all the text in this section of the results for better reading.

**Recommendations for the authors:**

**Reviewer #1 (Recommendations for the authors):**

(1) The cause and timing of lethality in the homozygous Wac knockout should be reported or discussed. Investigating Wac homozygous knockout embryos, if viable at early stages, could provide valuable insight into the developmental origins of the neuroanatomical and behavioral phenotypes described in the heterozygous animals. Even a brief histological or transcriptomic characterization of embryonic brains would strengthen the mechanistic understanding of Wac function during neurodevelopment.

We agree and have collected embryos as early as embryonic day 12.5 from multiple litters but never detected a knockout. We have added this text to the animal methods sections to let readers understand effort had been done to determine when death occurs. While we don’t currently explore this further in mice we now include zebrafish waca; wacb double knockouts. Notably, while we were able to generate a few of these mutants, most died. However, some zebrafish were aged long enough to observe lethal deficits in heart formation and swim bladder development, suggesting that early loss of Wac could impact these critical organs that leads to death.

(2) A better description of the data reported in Supplementary Tables 3 through 5 is needed. Supplementary Table 3 does not report any statistically significantly differentially expressed genes in the FDR column, and Supplementary Table 5 reports only two, and the reader should understand what the columns are indicating.

We have now added figure legend text to the supplementary file to explain each Table mentioned here.

**Reviewer #2 (Recommendations for the authors):**

(1) Page 3, last paragraph. The description of wacb is confusing. I recommend that the authors provide the unshown data they mention and also further explanation of the breeding scheme and result. Indeed, if wacb is homozygous lethal, does that make it more like the mouse WAC gene, and thus potentially the more relevant paralogue to study? Are both waca and wacb expressed in the same tissues? How does that compare to mouse and human WAC expression? Such figures about gene expression (even when adapted with permission from public resources like Allen brain atlas or GTEX) are common in this sort of paper, as they can be helpful to understand when and where the gene is thought to act. For waca vs. wacb, they may help determine which gene is more relevant to the brain (for example, if only one is expressed in the brain).

First, this is a great question and we have now added whole mount in situ for the waca and wacb genes as Figure S1. These data show low to no wacb expression in brain regions while waca is highly expressed there. Since the waca mutants showed phenotypes relevant to DESSH but wacb mutants did not, this correlates with observed expression patterns without fully excluding wacb from any role. Thus, we also made waca/wacb double KO zebrafish that showed a lethal phenotype, similar to homozygous mice mutants. Only a few waca; wacb double knockouts survived a little through development and are now shown in Figure S1. Since wacb KO zebrafish did not show any detectable phenotype on their own, we did not include the data since there are already several figures/tables in this manuscript. However, the waca KO zebrafish did show phenotypes similar to humans with DESSH and are the ones we focused on.

(2) Why did the authors cross the mice into the outbred CD1 background? Usually, most labs keep the lines on an inbred background. Was there a particular rationale here? I am not saying that they could not outcross them. It is just a bit puzzling why. Perhaps a sentence of explanation in the methods section would be warranted.

This is a great question and we have now added text to the animal methods section. Many labs that study development, especially on genes critical for survival/life like the Wac gene, use a more robust strain like CD-1. By doing this, we have a better chance of evaluating mutants at more mature ages and getting enough progeny to do more reproducible studies.

(3) A typical first experiment in a new knockout (fish or mouse) is to establish that the deletion does indeed result in a loss of RNA and protein. In the absence of this, the rest of the paper cannot be as confidently interpreted.

We did this for the mouse model and found reduced protein expression in the constitutive Het, however this datum is part of the western blots in figure 5. We now mention this in the early results section that protein levels were reduced in the Hets but maintain that the presentation of the western blot is better suited in Fig. 5 to compare to the other western blots. For zebrafish this was attempted but was more difficult. Available antibodies don’t work in zebrafish. RNA expression was attempted in both models and due to Wac being a critical gene for life, there are checks in place to upregulate faulty and normal RNA in the waca model. We screened for frameshift mutations in multiple KO lines and confirmed it by genomic DNA sequencing. In making many KOs and large-scale mutagenesis in zebrafish, we usually depend on phenotype-genotype segregation in Mendelian inheritance for many generations.

(4) Are these new lines indeed knockouts? I did find a WAC western as part of a later figure for the mouse. The authors may want to mention that earlier, or present at least that data right away. What about in the fish? Is there a way to confirm at the RNA or protein level that it is indeed a null allele?

Yes, as mentioned in the above response we have now mentioned our Wac western blot results early when introducing the mouse mutants and the issues with doing this in fish are presented above as well.

(5) Why are fish used that are KO while mice are Hets? Are WAC homozygous mice not viable? This should be mentioned. Regardless, the rationale for examining heterozygous mice and homozygous mutant fish should be provided. Each kind of experiment is useful, but they are interpreted in different ways. Hets will genocopy the patients, who are generally hets, while KOs are often useful for a study of the essential roles of the genes, even if they are not really modeling the patient gene dose.

Wac homozygous mice in our hands are embryonic lethal, now mentioned in the animal methods section, but we found early on that the Hets mimic several human DESSH patients. In zebrafish it is more complicated. We analyzed waca and wacb hets in zebrafish but found no phenotypes. This could be in part due to some complementation between the waca and wacb genes. It is also possible that a full waca KO could resemble a human DESSH individual since wacb may complement somewhat, even though deleting wacb entirely does not have a measurable phenotype. We have added more text to the discussion to explore these complexities. We also made waca/wacb double KO (dKO) zebrafish but they showed lethal phenotype, similar to homozygous mice mutants and suggesting some complementation by the wacb gene even though alone it did not exhibit phenotypes.

(6) Figure 3A: It does not appear that the authors are directly statistically comparing the two groups (genotypes) that they are drawing conclusions about. This is an unfortunately common mistake in the neuroscience literature across papers. There is a nice older review about it here. https://pubmed.ncbi.nlm.nih.gov/21878926/. To draw conclusions about the differences between the mouse genotypes, they need to compare the two genotypes directly with a statistical test. See Nygard et al for a recommended approach, like comparing social preference indexes

(https://onlinelibrary.wiley.com/doi/abs/10.1002/aur.2154).

Thank you for this information. Previous reviewers at a different journal asked for this particular evaluation. We have now made changes to address the assessment, and graphs now reflect comparisons of genotypes instead of a single genotype between time with a mouse or object. We have also moved to using a social discrimination index to compare the genotypes, similar to the study mentioned.

(7) MRI - it is a bit weird to separate the male and female brains just for the MRI. Was there a premise from human data to do so? If not, the authors should probably pool them. If they are concerned there are sex effects (or, more likely, a sex by genotype interaction) I recommend that they use a two-factor ANOVA and simply put both sex and genotype into the model. This will also have the advantage of increasing their statistical power for genotype effects a bit. If their current results are robust, they will still show up as a significant sex x genotype interaction.

All data in the manuscript initially compared the sexes to each other. We have now added this text to the animal section of the methods: For MRI, some zebrafish behaviors and now the RNA-seq data, sex was a difference and due to this observation, sex was (or now is) presented independently for these measurements. We now state that if no sex differences were observed the data were pooled.

(8) Also, did the authors correct for multiple testing in the MRI analysis? Since they are testing many regions, there is a risk of false positives if they do not. This could be confounded further by their splitting the data by sex, thus doubling the number of tests.

As noted above we did not do multiple corrections given the large number of regions and low number of replicates.

(9) How many images per animal were analyzed for the cell counts? This detail is absent from the methods and would help with evaluating the robustness of these findings. What other approaches were used to make sure the counting was unbiased?

We analyzed 3-4 images per animal for counts and counted hundreds of cells per image. In addition, the person counting was blinded to avoid any bias. These details have now been updated in the methods.

(10) As with the MRI, for the DEG analysis, I recommend the authors simply put sex and genotype into the same model as two factors (with an interaction), to increase their sensitivity to genotype effects, as well as be able to report on robust genotype x sex differences, if there are any. They may also consider testing the model with and without excluding the three outlier animals on their PCA. It may be that the noise of those outliers is detracting from their sensitivity for DEGs somewhat.

We greatly expanded our analyses and found more robust and unique changes in males that are now added to Figure 7 and supplemental files. After considering the data, decided to highlight the sex differences separately.

(11) A few more relatively simple things could readily be done with the RNAseq data to add some depth and interpretation. For example, do the hits here overlap other published IDD/autism DEG lists from mouse knockouts studies of genes like FoxP2, Chd8, Dnmt3a, Myt1l, Tcf4, etc? Do autism genes show up in the lists of hits here? And if so, more than expected by chance? Can they provide some visualization of their GO results in the main figure?

When we looked into the sex differences more we found that only the males showed significant upregulation of other autism risk genes increase that was previously unappreciated when the sexes were assessed together. Yes, several autism genes do show up but is heavily biased to males. Our main Figure 7 and new supplemental files show new GO term analyses and provide additional data looking not only autism but other factors.

(12) It appears the IMPC has phenotyped this mouse somewhat, including craniofacial abnormalities. They also report on some blood cell differences. Anyway, if no one has written about that data yet (as it was generated in the context of a big consortium effort), their guidelines may allow you to include some of their data as Supplementary Figures here with proper attribution. It might help to at least summarize useful findings from there in your discussion.

Due to the large number of figures/tables already in this report we don’t think this will be helpful. However, we do refer readers to the consortium in the animal methods section so they can explore data already generated by the IMPC.

(13) Minor/Typos:

(a) Figure 2K: I am confused by the description of three genotypes in the legend, but only two in the panel?

Corrected.

(b) I found it a little distracting that some results figures were embedded in the introduction.

We have moved the figures further in the manuscript to start in the results section.

(c) I don't understand this sentence: "Due to reduced sample size, sex-stratified DE was performed without model corrections at FDR < 0.1, 7 and found genes significantly upregulated and downregulated, respectively;" The sample size here seemed robust, so I am not sure what they were referring to? Are there missing numbers form this sentence? What is the 7? I think there are enough typos here that I am not sure how to evaluate this claim. Thus, the writing and clarity of this part could be improved.

This section had several typos that have now been corrected.

(d) "Marwan Shinawi, (unpublished results)" is a bit atypical of a citation. Are these results being reported with his permission? If so, then it should say 'personal communication' (if the journal permits this - some do not). If not, they should not report someone else's unpublished results without their explicit permission. It might upset some people to have their results presented this way.

We have changed unpublished results to personal communication. Marwin Shinawi is an author on this manuscript and has approved of everything we have reported.

(e) In all figures, consider shape or color coding for sex, even when pooling the data (e.g, the data points in the behavior figures).

This is a good idea but since we found no difference when analyzing the data we don’t see how this extra work will make a difference. Since we now mention that sex differences were only presented as separate graphs when observed in the methods we think this should be acceptable.
